# Alternative splicing of NF-YA promotes prostate cancer aggressiveness and represents a new molecular marker for clinical stratification of patients

**DOI:** 10.1186/s13046-021-02166-4

**Published:** 2021-11-15

**Authors:** Silvia Belluti, Valentina Semeghini, Giovanna Rigillo, Mirko Ronzio, Daniela Benati, Federica Torricelli, Luca Reggiani Bonetti, Gianluca Carnevale, Giulia Grisendi, Alessia Ciarrocchi, Massimo Dominici, Alessandra Recchia, Diletta Dolfini, Carol Imbriano

**Affiliations:** 1 Department of Life Sciences, University of Modena and Reggio Emilia, via Campi 213/D, Modena, Italy; 2 Department of Biosciences, University of Milan, Milan, Italy; 3 Centre for Regenerative Medicine, Department of Life Sciences, University of Modena and Reggio Emilia, Modena, Italy; 4 Laboratory of Translational Research, Azienda Unità Sanitaria Locale-IRCCS di Reggio Emilia, Reggio Emilia, Italy; 5 Department of Medical and Surgical Sciences for Children & Adults, Division of Pathology, University-Hospital of Modena and Reggio Emilia, Modena, Italy; 6 Surgical, Medical and Dental Department of Morphological Sciences Related to Transplant, Oncology and Regenerative Medicine, University of Modena and Reggio Emilia, Modena, Italy; 7 Laboratory of Cellular Therapy, Program of Cell Therapy and Immuno-Oncology, Division of Oncology, Department of Medical and Surgical Sciences for Children & Adults, University-Hospital of Modena and Reggio Emilia, Modena, Italy

**Keywords:** NF-Y, Prostate cancer, Alternative splicing, Genome editing, Transcriptome profiling

## Abstract

**Background:**

Approaches based on expression signatures of prostate cancer (PCa) have been proposed to predict patient outcomes and response to treatments. The transcription factor NF-Y participates to the progression from benign epithelium to both localized and metastatic PCa and is associated with aggressive transcriptional profile. The gene encoding for NF-YA, the DNA-binding subunit of NF-Y, produces two alternatively spliced transcripts, NF-YAs and NF-YAl. Bioinformatic analyses pointed at NF-YA splicing as a key transcriptional signature to discriminate between different tumor molecular subtypes. In this study, we aimed to determine the pathophysiological role of NF-YA splice variants in PCa and their association with aggressive subtypes.

**Methods:**

Data on the expression of NF-YA isoforms were extracted from the TCGA (The Cancer Genome Atlas) database of tumor prostate tissues and validated in prostate cell lines. Lentiviral transduction and CRISPR-Cas9 technology allowed the modulation of the expression of NF-YA splice variants in PCa cells. We characterized 3D cell cultures through in vitro assays and RNA-seq profilings. We used the rank-rank hypergeometric overlap approach to identify concordant/discordant gene expression signatures of NF-YAs/NF-YAl-overexpressing cells and human PCa patients. We performed in vivo studies in SHO-SCID mice to determine pathological and molecular phenotypes of NF-YAs/NF-YAl xenograft tumors.

**Results:**

NF-YA depletion affects the tumorigenic potential of PCa cells in vitro and in vivo. Elevated NF-YAs levels are associated to aggressive PCa specimens, defined by Gleason Score and TNM classification. NF-YAl overexpression increases cell motility, while NF-YAs enhances cell proliferation in PCa 3D spheroids and xenograft tumors. The transcriptome of NF-YAs-spheroids has an extensive overlap with localized and metastatic human PCa signatures. According to PCa PAM50 classification, NF-YAs transcript levels are higher in LumB, characterized by poor prognosis compared to LumA and basal subtypes. A significant decrease in NF-YAs/NF-YAl ratio distinguishes PCa circulating tumor cells from cancer cells in metastatic sites, consistently with pro-migratory function of NF-YAl. Stratification of patients based on NF-YAs expression is predictive of clinical outcome.

**Conclusions:**

Altogether, our results indicate that the modulation of NF-YA isoforms affects prostate pathophysiological processes and contributes to cancer-relevant phenotype, in vitro and in vivo. Evaluation of NF-YA splicing may represent a new molecular strategy for risk assessment of PCa patients.

**Supplementary Information:**

The online version contains supplementary material available at 10.1186/s13046-021-02166-4.

## Background

Prostate cancer (PCa) is characterized by high clinical heterogeneity and multiple levels of molecular signatures [1]. Despite the predominant luminal-like phenotype observed in human patients [2], PCa seems to originate by both basal and luminal cells, the two main epithelial lineages of prostate gland. Transgenic mouse models have been used to understand the origin of PCa: basal- and luminal-origin tumors in inducible Pten-inactivated mice exhibited similar histopathology but significant different molecular signatures [3]. In particular, genes overexpressed in luminal-origin tumors were enriched in a lethal PCa signature generated by high-throughput expression profiling of prostate samples from a watchful waiting patient cohort [4], consistently with a more aggressive phenotype of luminal-origin tumors. Independent transcriptomic studies identified three PCa molecular subtypes: PCS1/Luminal B (LumB) tumors, which show high proliferation, poor differentiation and prognosis, PCS2/Luminal A (LumA) differentiated tumors with lipid and steroid biosynthetic signature, and PCS3/basal-cell-like subtype, characterized by extracellular matrix (ECM) organization, inflammation and cell migration genes expression [5, 6].

The binding site for the heterotrimeric transcription factor (TF) NF-Y has been identified as biologically relevant within the molecular network of genes up-regulated during PCa progression, from benign epithelium to localized and hormone-refractory metastatic PCa [7]. These data perfectly agree with the over-representation of the NF-Y-binding motif, the CCAAT box, in promoters of genes overexpressed in multiple types of cancers [8]. Moreover, studies on patients samples highlighted a correlation between the up-regulation of NF-Y subunits, NF-Y-target genes, cancer aggressiveness and poor prognosis (for a review see [9]).

The gene encoding for the NF-YA DNA-binding subunit undergoes alternative splicing: skipping or inclusion of the second coding exon (exon 3) gives rise to NF-YA proteins with different molecular weights, namely NF-YA short (NF-YAs) and NF-YA long (NF-YAl) [10]. The two proteins share the NF-YB/NF-YC interaction and the DNA binding C-terminal domains, while they differ in the length of the N-terminal Glutamine-rich transactivation domain, with NF-YAl being 28/29 aa longer than NF-YAs. Tissue- and cell-type specificity of the expression of the two isoforms has been clearly demonstrated [10, 11]. While in vitro assays suggested that the two isoforms have equal transcriptional activity [10, 11], recent reports have demonstrated that NF-YAs and NF-YAl are not functionally equivalent [12–15]. In particular, NF-YAs levels increase at the expense of NF-YAl in all subtypes of lung adenocarcinomas (LUAD) [16] and in the majority of patients tissues of lung squamous cell carcinomas (LUSC), with NF-YAs^high^ subtypes being characterized by cell-cycle transcriptional signature and NF-YAl^high^ tumors by a pro-migration transcriptional profile [17]. Similarly, in breast cancers the ratio between NF-YAs/NF-YAl isoforms shifts towards NF-YAs, with the exception of aggressive and metastasis-prone basal-like Claudin^low^ tumors, which show high expression of NF-YAl together with typical epithelial to mesenchymal transition (EMT) markers [18]. Finally, while NF-YAs is undetectable in benign endometrial tissues, it is consistently expressed in high grade type I endometrial cancer (EC). Moreover, in low grade ECs, the presence of NF-YAs associated to low Lamin A levels is predictive of poorly differentiated tumors and poor prognosis [19].

Altogether, these studies pointed at NF-YA splicing as a key transcriptional signature that can improve patients’ stratification and better discriminate between different molecular subtypes. Likewise, here we show that NF-YA isoforms are differently expressed in PCa with respect to healthy prostate epithelial tissue and drives the formation of tumors with different phenotypes. Modification of the expression of NF-YA splice variants, through lentiviral expression and CRISPR-Cas9 technology in PCa cells, demonstrates that the two isoforms have distinct roles in viability, proliferation and invasion both in vitro and in vivo. The two NF-YA isoforms regulate different transcriptional cancer-associated programs, with NF-YAs-associated transcriptome overlapping with both localized and metastatic PCa. Our study demonstrates the different contribution of the two NF-YA isoforms in PCa progression and aggressiveness and highlights the potential use of NF-YAs for risk stratification in PCa patients.

## Methods

### TCGA data analysis

RNA-seq and clinical data of TCGA- Prostate Adenocarcinoma (PRAD) dataset were retrieved from firebrowse.org. The cohort was made of 498 tumor samples and 52 normal samples. Survival data were downloaded from UCSC Xena repository [20]. PAM50 classification on TCGA data were extracted from PCTA database for 153 tumor samples (http://www.thepcta.org/about/) [6], whereas the classification of remaining samples was predicted employing a deep learning based classification framework, DeepCC [21].

PCS classification of samples was determined according to a highly specific 37 genes panel signature for PCS1, PCS2 and PCS3 [6]. Genes z-scores were calculated and for each PCS signature a median z-score was computed. Each sample was assigned to the PCS corresponding to the highest median z-score. RNA-seq raw data of metastatic castration-resistant prostate cancer (Met. CRPC) samples and Circulating Tumor Cells (CTCs) were retrieved from Geodata, accession codes GSE147250 and GSE104209 respectively. Both datasets were aligned against human transcriptome (GRCh37/hg19) using bowtie2 wrapper within RSEM 1.3.1 tool.

To compare gene expression of CRPC samples and CTCs, raw counts of both datasets were normalized with DESeq2 algorithm [22] and the Variance Stabilizing Transformed (vst) counts were used. As TCGA RNA-seq data employ a different gene annotation from Met. CRPC and CTCs datasets, DESeq2 normalization was not applicable. Hence, we considered the ratio of NF-YA isoforms and we obtained box plots comparing processed TCGA RNA-seq data with the Met. CRPC dataset after log2 transformation of TPM and z-score normalization.

Box plots were obtained using ggplot2 R package and significance of comparison was estimated with Wilcoxon test. Survival analyses were performed employing survminer R package, where *p* values were computed according to the log-rank test. Cox proportional hazard regressions were computed with the finalfit R package. The analyses have been performed using R (v 4.0.5).

### Cell lines and lentiviral transduction

Normal PNT1A (ECACC Cat# 95012614, RRID:CVCL_2163) were grown in RPMI 1640 Medium and Ham’s F12 Medium (1:1). Cancer DU145 (ATCC Cat# HTB-81, RRID:CVCL_0105) and LNCaP (ATCC Cat# CRL-1740, RRID:CVCL_1379) were grown in RPMI 1640 Medium. Cancer PC3 cells (ATCC Cat# CRL-1435, RRID:CVCL_0035) were maintained in Ham’s F12 Medium. All media (Biowest) were supplemented with 2 mM glutamine, 100 IU/ml penicillin, 100 μg/ml streptomycin and 10% FBS (Gibco). Normal RWPE-1 (ATCC Cat# CRL-11609, RRID:CVCL_3791) were grown in Keratinocyte Serum Free Medium (K-SFM; # 17005042, Gibco), supplemented with 0.05 mg/ml bovine pituitary extract (Gibco) and 5 ng/ml human recombinant epidermal growth factor (Gibco). Cell lines were authenticated by Short Tandem Repeat (STR) Analysis (Eurofins Genomics), verified to be mycoplasma free and passaged < 3 months. Healthy N1 cells and benign prostate hyperplasia C10 and C17 cells were a kind gift of Dr. A. Farsetti [23] and were cultured in IMDM Medium (Biowest) supplemented with 10% FBS (HyClone). All cells were grown at 37 °C in a humidified incubator containing 5% CO2.

NF-YA inactivation was obtained by lentiviral infection of PC3 cells with pLKO.1/EGFP non-targeting shRNA (shCTR) or shRNA targeting exon 6 of NF-YA (shNF-YA), previously described [24]. Cells were harvested 72 h post-infection (MOI = 20).

Stable NF-YA-overexpressing cell lines were obtained by lentiviral infection of PC3, LNCaP or DU145 cells with either pSIN-NF-YAs, pSIN-NF-YAl or control pSIN-Empty particles and puromycin selection [13].

### Anchorage-dependent and anchorage–independent colony assays

Colony formation assays were performed as previously described [25]. For anchorage-dependent clonogenic assay, 1000 cells were seeded in 6 wells plates for 7 days and colonies were fixed and stained with 0.5% crystal violet solution in 20% Methanol. For anchorage-independent colony formation assay, 2000 cells were resuspended in 0.25% agarose in complete cell growth medium and plated on top of a base layer (0.6% agarose). After 3 weeks (PC3) or 4 weeks (LNCaP) colonies were stained with 0.07% crystal violet solution in 1X PBS. Plates were imaged and colonies were counted with automated colony counter (OpenCFU 3.9.0).

### Generation and culture of Multicellular Tumor Spheroids (MTSs)

5000 PC-3 cells were plated into 96-well Round Bottom Ultra-Low Attachment (ULA) plates (#7007 and #4515, Corning) in 150 μl complete ice-cold medium containing 0.5 mg/ml Matrigel matrix (#354248, Corning) followed by centrifugation for 10 min at 1000×g, 4 °C, using no braking. 2000 LNCaP cells were seeded into 96-well Round Bottom ULA plates in 200 μl complete medium and centrifuged at 1000×g, 10 min. DU145 cells were seeded into 96-well Round Bottom ULA plates in 200 μl complete medium containing 0.67 mg/ml Matrigel matrix. MTSs were incubated for 7 days (PC3), 14 days (LNCaP) or 10 days (DU145) at 37 °C, 5% CO2 in humidified incubators. Fifty microlitres of fresh spheroid medium was added every 4 days. MTSs images were acquired with an EVOS M5000 imaging system (Thermo Fisher Scientific) and analysed with ImageJ software for manual and automated measurements of projected areas [26].

### Analysis of cell proliferation and viability

Cell viability in transduced shCTR/shNF-YA cells was measured by colorimetric 3-(4,5-Dimethylthiazol-2-yl)-2,5-diphenyltetrazolium bromide (MTT) assay [25].

Cell viability of MTSs was assessed using LIVE/DEAD imaging. Spheroids were incubated for 1 h in PBS containing 1 μM Calcein-AM (#80011, Biotium) and 2 μM Propidium Iodide (PI). The samples were then imaged with an EVOS M5000 fluorescence microscope (Thermo Fisher Scientific) with a 4X objective and a Nikon A1 confocal laser scanning microscope.

Cell proliferation in MTSs was assessed by cytofluorimetric analysis. Spheroids were cultured for 7 days and 20 μM BrdU was added for the final 16 h. Single cell suspension was then obtained by incubation in warm 0.25% Trypsin-EDTA, at 37 °C, for 10 min, pipetting up and down 10 times every 3 min and 30 times at the end of incubation, to help dissociation. After washing with 1X PBS/0.5% Tween-20, cells were treated with 2 N HCl for 30 min at RT, followed by the addition of 0.1 M borate buffer (pH 8.5) for 5 min, RT. Cells were then washed and incubated with mouse anti-BrdU antibody (#347580, BD Biosciences) for 1 h at 4°C. After washing, cells were incubated with FITC anti-mouse secondary antibody (#F0313, Agilent Dako) for 1 h at 4C, washed in 1X PBS and resuspended in citrate solution (Na–Citrate 3.4 mM, NaCl 9.65 mM, NP-40 0.03%). Apoptotic cells were stained with AnnexinV-FITC (#V13242, Invitrogen), according to the manufacturer’s protocol. BrdU-positive and AnnexinV-positive cells were analyzed by an Attune Nxt cytofluorimeter (Thermo Fisher Scientific).

### Whole-mount Immunostaining of MTSs

MTSs were carefully handled with cut P200/P1000 pipette tips and entire procedure was performed in suspension, allowing MTSs to settle under gravity on ice, to preserve morphology. MTSs were washed with 1X PBS and fixed in 10% Formalin Solution (#HT5014, Sigma-Aldrich) for 20 min, RT. After washing with 1X PBS, MTSs were permeabilized with ice-cold 1X PBS/0.5% TritonX-100 for 10 min at RT and incubated 10 min in blocking buffer (3.5% BSA, 0.1% TritonX-100 in 1X PBS). MTSs were incubated over-night at 4 °C with anti-Ki-67 (dilution 1:300 in blocking buffer; #9129, Cell Signaling). After two washes with 1X PBS for 5 min, Alexa-fluor 488-conjugated antibody (dilution 1:400 in blocking buffer; #A-21206, Thermo Fisher Scientific) was incubated for 2 h at RT, followed by nuclei staining with DAPI (1:5000 in 1X PBS) for 10 min. After washing with 1X PBS, MTSs were mounted on slides with imaging spacers in Mowiol. Images were acquired using an EVOS M5000 (Thermo Fisher Scientific) fluorescence microscope.

### RNA-seq and gene expression analysis of MTSs

Eighteen MTSs per cell line were pooled and RNA for sequencing was extracted by automatic extractor MaxwellRSC (simply RNA Cells kit, Promega). Three independent experiments were performed. RNA-seq libraries preparation and sequencing were performed as previously described [27]. RNA-seq raw data were aligned against human transcriptome (GRCh37/hg19) using bowtie2 wrapper within RSEM 1.3.1 tool. Significant differential expressed genes (DEGs) were computed with DESeq2 setting FDR < 0.01 and |Log2FC| > 1 in R environment. Unique DEGs for one comparison, e.g. Short vs Empty, were obtained starting from its total DEGs and filtering out genes shared with the other comparison, e.g. Long vs Empty, having |Log2FC| > 0.5. Gene Ontology enrichment on DEGs was performed using KOBAS 3.0 (http://kobas.cbi.pku.edu.cn/anno_iden.php) and top 25 terms with FDR < 0.001 were kept. More general GO terms, namely those with background number of genes higher than 500, were discarded. Rank-Rank Hypergeometric Overlap (RRHO) was performed with RRHO2 R package by ranking Log2FC of all differential expressed genes for each comparison.

### Migration and invasion assays

The migration and invasion assays were performed with Transwell membranes (pore size 8 μm; #3464, Corning) without coating (migration assay) or coated with 50 μl of 1 mg/ml Matrigel (#354248, Corning) in serum-free medium (invasion assay). PC3 cells were starved overnight and 5 × 10^4^ cells were seeded in 100 μl of serum-free medium into the upper chamber of the transwell. Complete F12 medium containing 10% FBS was used as chemoattractant in the lower chamber. After 18 h (migration assay) or 26 h (invasion assay), the cells were removed with a cotton swab from the upper chambers, while cells on the underside of the inserts were fixed and stained with 0.5% crystal violet solution in 20% Methanol, for 15 min. Five randomly selected fields were counted under a light microscope and percent migration/invasion was calculated according to the manufacturer’s protocol.

For wound healing cell migration assay, PC3 cells were seeded into Ibidi culture-insert (#80209, Ibidi GmbH). Cells were cultured for 24 h and then the culture-insert was removed to create the gap. Cells were gently washed with 1X PBS and complete growth medium was provided. Images were acquired immediately after removal of the insert and over 22 h. Wound areas were measured using Photoshop software and residual wound area (%) was calculated with the formula: (final area/initial area × 100).

3D spheroid invasion assays were performed in (i) growth factors–enriched conditions and (ii) chemotactic conditions: (i) spheroids were assembled by plating 2000 PC3 cells in 96-well Round Bottom ULA plates (#4515, Corning). After 3 days, Matrigel matrix (#354248, Corning) was added to each well to 1 mg/ml final concentration. Optical microscopy images were taken after 7 days and the area (A) of whole and core spheroids was calculated with Photoshop software. Spheroid invasion index (%) was calculated with the formula: (A_tot_ - A_core_)/A_tot_ × 100. (ii) MTSs were generated by centrifugation of 5000 PC3 cells into 96-well Round Bottom ULA plates (#7007 and #4515, Corning), in complete growth medium containing 0.5 mg/ml Matrigel matrix (#354248, Corning), as described above. After 7 days, MTSs were gently collected and washed with 1X PBS, using cut P200/P1000 pipette tips to preserve morphology. MTSs were embedded into 5 mg/ml Growth Factor Reduced Matrigel (#354230, Corning) diluted in F12 medium containing 1% FBS. One 50 μl drop containing one MTS was dispensed in a 24-well plate and allowed to polymerize into a 37 °C incubator. Embedded spheroids were then submerged in F12 warm culture medium containing 10% FBS as chemoattractant. Invasion was assessed after 7 and 11 days by optical microscopy with an EVOS M5000 imaging system (Thermo Fisher Scientific).

### In vivo mouse xenograft study

Male SCID Hairless Outbred (SHO) mice (Strain Code #474, Charles River Laboratories) were kept under protocols approved by the Institutional Animal Care Committee and by National Institute of Health (Ministero della Salute) (n. 709/2017-PR). Six groups of mice were established: transduced shCTR and shNF-YA PC3 cells (*n* = 7/each), stable PC-3 Empty, NF-YAl, NF-YAs (*n* = 8/each) and NF-YAs-only (*n* = 6). 5 × 10^5^ PC3 cells mixed at a 1:1 dilution with Matrigel HC (#354248, Corning) were sub-cutaneously flank-injected: the length and width of the xenografts were measured once a week with a digital caliper and tumor volumes were estimated using the formula V = 0.5 × a × b^2^, where a and b are the largest and smallest diameters, respectively. The rate of growth was calculated using the following formula: (Vol 2-Vol 1)/Vol 1 × 100%, where Vol2 = tumor volume on measured week; Vol1 = tumor volume on previous week [28]. After 5 weeks, animals were sacrificed and tissues were harvested. The tumor specimens were divided into two parts, one for snap freezing in liquid nitrogen for protein/RNA extraction, the other was fixed in 10% formalin solution for 24 h and embedded in paraffin for histopathological analyses. Lung samples were collected and fixed in 10% Formalin Solution (#HT5014, Sigma-Aldrich) for 24 h at 4 °C and frozen.

### Histology and immunohistochemistry (IHC) on FFPE sections

Histopathology and IHC evaluation were performed by a pathologist on formalin-fixed and paraffin-embedded (FFPE) tumor sections. For histological analysis of tumor spheroids, handling of MTSs was carefully performed with cut P200/P1000 pipette tips, to preserve morphology. MTSs were fixed in 10% Formalin Solution (#HT5014, Sigma-Aldrich) over night at 4 °C, washed in PBS and embedded in 1% agarose gel in Peel-A-Way embedding molds (#E6032, Sigma-Aldrich). The thin agarose blocks containing MTSs were dehydrated and paraffin embedded. Four micrometres FFPE sections were prepared and stained with standard Haematoxylin and Eosin (H&E). For analysis of xenograft tumor tissues, 3,5 μm-thick sections were prepared and stained following standard H&E automatic procedure routinely adopted by the Anatomic Pathology laboratory (Leica Biosystems). Immunohistochemical analysis of Ki-67 (MIB1 clone, dilution 1:100; #M7240, Dako) was performed using a Bench Mark ULTRA automated stainer (Ventana Medical Systems). Ki-67 label index was measured by pathologist visual scoring, as the percentage of tumor cells immunoreactive for Ki-67 over the total number of tumor cells. Mean values were obtained by scanning overall tumor masses, observed under 40X optical microscope.

### Protein extraction and Immunoblotting

Whole-cell protein extracts were prepared by lysis of 2D cultured cells into 1X SDS sample buffer (25 mM Tris–HCl pH 6.8, 1.5 mM EDTA, 20% glycerol, 2% SDS, 5% b-mercaptoethenol, 0.0025% Bromophenol blue). Protein lysates of MTSs were obtained from a pool of at least eight spheroids, through a two step lysis procedure: MTSs’ proteins were extracted in RIPA buffer (50 mM Hepes pH 7.9, 140 mM NaCl, 1 mM EDTA, 1% Triton X-100, 0.1% sodium deoxycholate, 0.1% SDS, 0.5 mM PMSF and protease inhibitor cocktail) for 30 min on ice and then one volume of 2X SDS sample buffer was added to the samples for complete lysis. Protein lysates from tumor xenografts were obtained in RIPA buffer with protease and phosphatase inhibitors, on ice, using a potter homogenizer. Homogenates were incubated on ice for 30 min, and then sonicated 2 min (in rounds of 10 s sonication/10 s rest for each cycle, on ice). Extracts were then cleared by centrifugation at > 10,000 x g for 30 min at 4 °C.

Equivalent amounts of cellular extracts were resolved by SDS-PAGE, transferred to PVDF membrane with Trans-Blot Turbo Transfer System (Bio-Rad) and immunoblotted with the following primary antibodies, diluted 1:1000 in 1X TBS with 1 mg/ml BSA: anti-NF-YA (#17753, Santa Cruz Biotechnology), anti-Tubulin (#66031, Proteintech Europe), anti-AKT (#MAB2055, R&D Systems) and anti-phospho-AKT(S473) (#AF887, R&D Systems). Membranes were blotted and scanned with an Amersham Imager AI680 RGB (GE Healthcare), using detection reagents Westar ηC and Supernova HRP substrates (Cyanagen).

### RNA extraction and RT-qPCR

Xenograft tumor tissues were lysed with Tri-Xtract™ reagent (#786–652, G-Biosciences) using a polytron homogenizer (POLYTRON® PT 10–35, Kinematica). RNA was further purified with Ribospin II mini Kit (#314–150, GeneAll), according to the manufacturer’s protocol. Two hundred nanograms of RNA were retrotranscribed with PrimeScript RT Reagent Kit (#RR037A, Takara Bio) and quantitative PCR was performed with SsoAdvanced Universal SYBR Green Supermix (#1725274, Bio-Rad), using a Biorad CFX Connect™ Real-Time PCR Detection System. Data were analyzed using the Bio-Rad CFX Maestro 2.0 software (Bio-Rad) and mRNA expression was normalized to RPS20 and bACTIN genes. Oligonucleotides are listed in Supplementary Table S1.

### Extraction of genomic DNA and Alu-qPCR

Genomic DNA was extracted from formalin-fixed mouse lungs using a heat and alkaline protocol previously described [29]. Briefly, lungs were minced and DNA extraction/reverse cross-link was performed in alkali digestion buffer (0.1 M NaOH, 1% SDS), at 100 °C for 40 min. DNA was purified and resuspended in TE buffer (10 mM Tris-HCl pH 8.0, 1 mM EDTA). Twenty nanograms genomic DNA was used in qPCR reactions, with highly sensitive and specific human Alu primers previously described [30] (Supplementary Table S1), in a total reaction volume of 20 μl with SsoAdvanced Universal SYBR Green Supermix (#1725274, Biorad). PCR was performed on a Biorad CFX Connect™ Real-Time PCR Detection System, under the following conditions: 1 cycle of 95 °C for 10 min, followed by 50 cycles of 95 °C for 15 s, 56 °C for 30 s and 72 °C for 30 s. qPCR threshold of detection was set so that the Cq values of negative controls (mouse DNA) or low amount of human genomic DNA outside the linear range of amplification were higher than this threshold value, set at 1 cycles below the Cq value of negative controls.

### CRISPR/Cas9 gene editing

To knockdown the expression of both long and short splicing variants of human *NF-YA* we employed the CRISPR/Cas9 system. sgRNA was designed to guide *Streptococcus pyogenes* Cas9 (SpCas9) to a 5′-TGG-3′ PAM sequence in exon 2 of the human *NF-YA* gene. To generate pX330.gRNA.PGK.GFP, we first cloned the designed sgRNA in the pX330-U6-Chimeric_BB-CBh-hSpCas9 plasmid (Addgene plasmid #42230; http://n2t.net/addgene:42230; RRID:Addgene_42,230) [31] by oligo annealing into BbsI sites (oligonucleotides are listed in Supplementary Table S1). Then, an expression cassette for GFP reporter gene under the control of the strong constitutive phosphoglycerate kinase promoter (PGK) was subcloned in pX330 plasmid downstream of the polyA signal of SpCas9 expression cassette. Control plasmid without gRNA was generated as described for effector plasmid.

NF-YAl or NF-YAs PC3 cells were transfected with either the effector or control plasmids using FuGENE HD Transfection Reagent (#E2311, Promega), with a plasmid: FuGENE HD ratio of 1:3.5, according to the supplier’s protocol. Cultures were visualized through fluorescence microscopy 48 h post-transfection and cells were then harvested in culture media at final concentration of 1x10^6^cells/ml: GFP positive cells were sorted by FACSARIA III flow cytometer (BD Biosciences) equipped with two air-cooled lasers at 488 and 633 nm wavelengths. Data were analysed by Diva software (BD Biosciences). Monoclonal cell populations were then isolated by limiting dilution into 96-well plates.

To analyze editing efficiency in treated cells, genomic DNA was extracted from CRISPR-treated NF-YAs or NF-YAl PC3 cells or clones, using QIAamp DNA mini or micro kits (Qiagen) following the manufacturer’s instructions. Human and mouse genomic regions flanking sgRNA target site were amplified by PCR using PCR GoTaq (Promega) (oligonucleotides are listed in Supplementary Table S1). Then, PCR amplicons were Sanger sequenced and analyzed by TIDE (Tranking of Indels decomposition, https://tide.nki.nl) software to evaluate the frequency of indels generated upon NHEJ-repair of CRISPR-mediated DSBs.

### Statistical analysis

All statistical analyses were performed with GraphPad PRISM 6 software (GraphPad Prism, RRID:SCR_002798), using unpaired t-test, multiple t-test, one-way ANOVA and two-way ANOVA with post-hoc tests, as specified in the figure legends. Graphs represent Means ± Standard Error of the Mean (SEM) and specific number of biological replicates (n values) are reported in each figure legend. Data are considered to be statistically significant if *p* < 0.05 (*), *p* < 0.01 (**), *p* < 0.001(***) and *p* < 0.0001 (****).

## Results

### Higher expression and altered splicing signature of NF-YA are associated to PCa aggressiveness

**Fig. 1:**
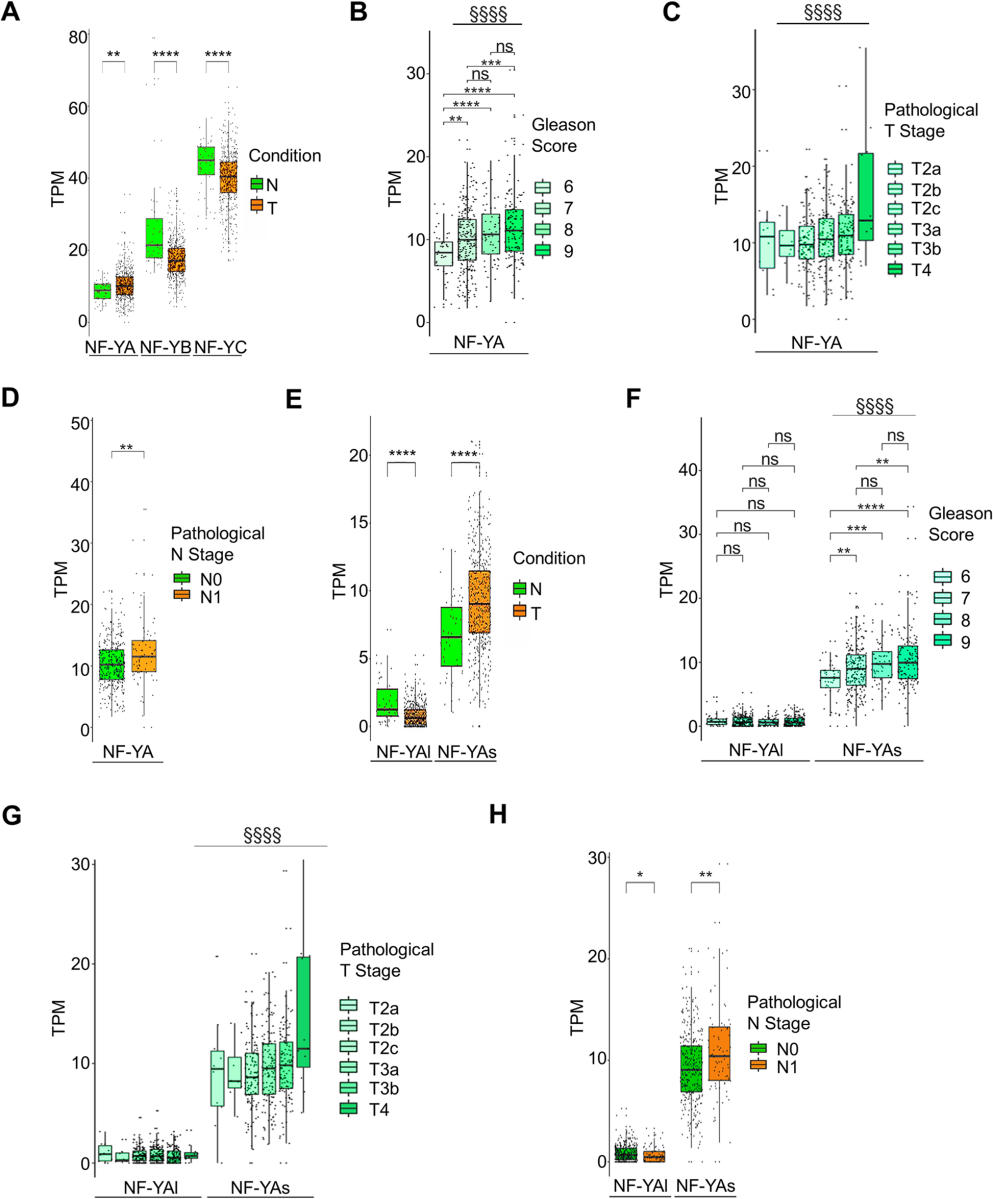
Expression of NF-YA and its splice variants in prostate cancer samples. **A** Expression levels of NF-YA, NF-YB and NF-YC measured as transcripts per million (TPM) in prostate adenocarcinoma (PRAD) patients compared to normal ones. N = normal samples, T = tumor samples. Wilcoxon test T vs N: **p < 0.01, ****p < 0.0001. **B** Transcript levels (TPM) of NF-Y subunits in PRAD patients according to Gleason Score stratification. Wilcoxon test T vs N: **p < 0.01, ***p < 0.001, ****p < 0.0001, ns, not significant. Jonckheere trend test: ^§§§§^ *p* < 0.0001. **C** NF-YA expression levels in PRAD samples stratified by pathological T stage. Jonckheere trend test: ^§§§§^ *p* < 0.0001. **D** NF-YA expression levels in PRAD samples stratified by pathological N stage. Wilcoxon test N1 vs N0: **p < 0.01. **E** Transcript levels (TPM) of NF-YAs and NF-YAl in PRAD patients compared to normal ones from TCGA data set. N = normal samples, T = tumor samples. Wilcoxon test T vs N: ****p < 0.0001. **F** TPM of NF-YAs and NF-YAl in PRAD patients according to Gleason Score stratification. Wilcoxon test: **p < 0.01, ***p < 0.001, ****p < 0.0001, ns, not significant. Jonckheere trend test: ^§§§§^ *p* < 0.0001. **G** Transcript levels (TPM) of NF-YA isoforms according to pathological T stage. Jonckheere trend test: ^§§§§^ *p* < 0.0001. **H** Transcript levels (TPM) of NF-YA isoforms according to pathological N stage. Wilcoxon test N1 vs N0: *p < 0.05, **p < 0.01

To identify whether global gene expression of NF-Y subunits is deregulated in PCa, RNAseq data were extracted from the TCGA (The Cancer Genome Atlas) database of tumor prostate tissues and compared to healthy ones. While NF-YA expression is significantly up-regulated in PCa samples, NF-YB and NF-YC subunits show decreased transcript levels (Fig. 1A). This opposite behavior is consistent with our previous results showing that a negative feedback loop controls the transcription of NF-Y subunits, with NF-YA down-regulation triggering NF-YB/NF-YC up-regulation and vice versa [32]. Therefore, we focused our attention on the NF-YA DNA-binding subunit, and we evaluated whether a correlation exists between NF-YA transcript levels and PCa grade and aggressiveness, determined by Gleason Score (GS). NF-YA expression proportionally raises with increasing GS (Fig. 1B). Moreover, NF-YA transcription is up-regulated in more aggressive pathological T (Tumor) stages, with T1 and T2 describing a organ-confined cancer, T3-T4 representing cancer spreading outside the prostate (Fig. 1C). We also identified that NF-YA increases in pathological N1 with respect to N0 stages, where N (Node) stage N1 means that the cancer has spread from the primary tumor to lymph nodes near the prostate (Fig. 1D).

As previously described, the NF-YA gene generates two spliced isoforms, which show different expression levels depending on tissue type, proliferation/differentiation and normal/tumor conditions [13, 14, 16–19, 33]. TCGA RNAseq data showed a significant increase in NF-YAs transcripts at the expense of NF-YAl in tumor prostate tissues compared to healthy ones (Fig. 1E). In particular, the increasing trend of NF-YAs showed a direct correlation with GS (Fig. 1F) and pathological T and N stages (Fig. 1G, H).

Altogether, these analyses suggested a possible contribution of NF-YA overexpression in PCa and pointed out a splicing signature associated to PCa progression, with NF-YAs being mostly expressed in more aggressive cancer types.

### NF-YA abrogation attenuates PCa-related cellular processes

**Fig. 2:**
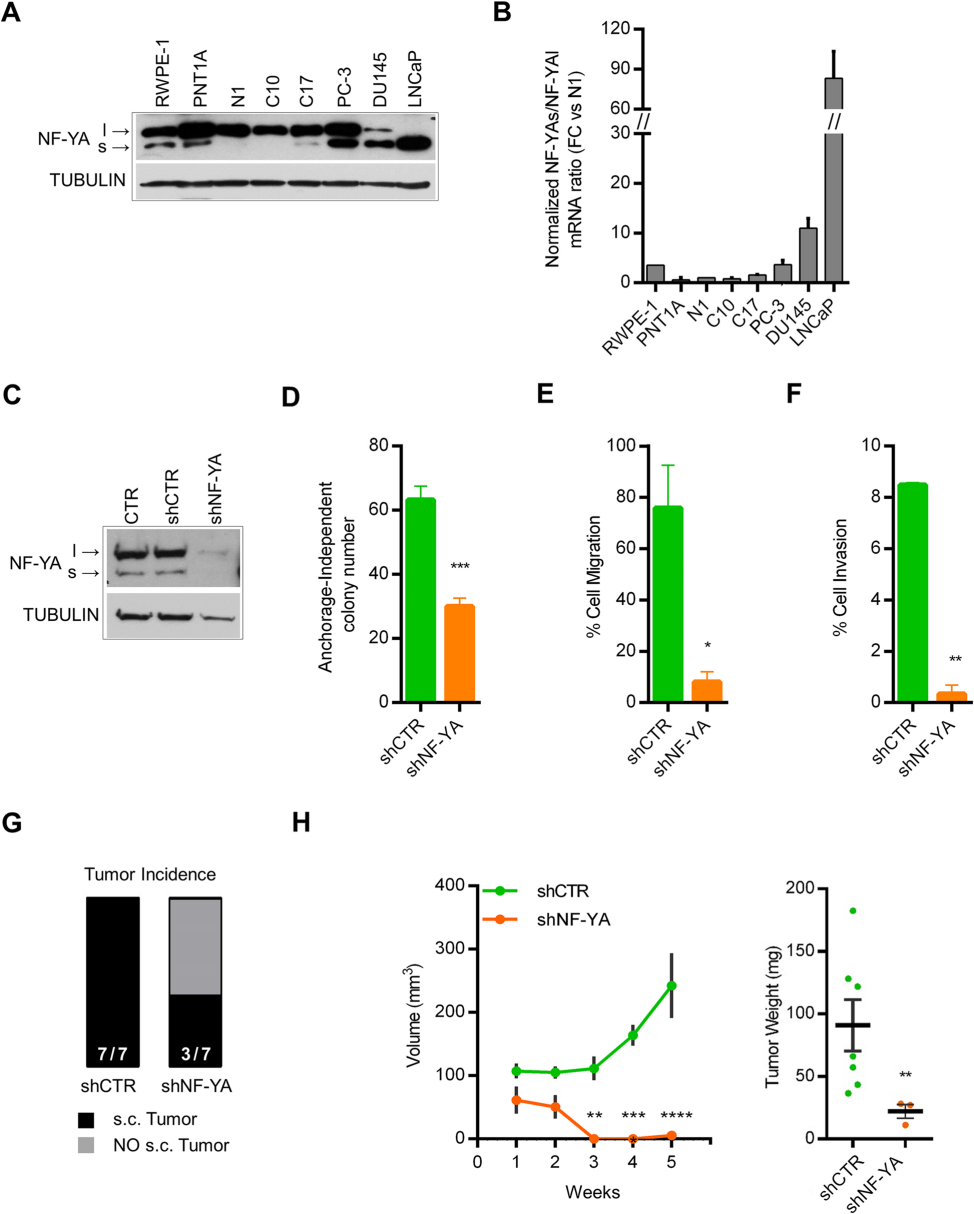
Effects of NF-YA inactivation in tumor prostate cells. **A** Expression of NF-YA splice variants in prostate epithelial normal and cancer cells by western blot analysis of total cellular extracts. N1 = primary healthy epithelial prostate cells, C10, C17 = primary BPH prostate cells. RWPE-1, PNT1A = normal immortalized cell lines. PC3, DU145, LNCaP = PCa cell lines. Tubulin was used as loading control of total cellular extracts. **B** Expression levels of NF-YA isoforms quantified by RT-qPCR and reported as fold change of NF-YAs/NF-YAl mRNA ratio vs N1 cell line levels, arbitrarily set at 1. Rpl21 was used as reference gene. **C** Western blot analysis of NF-YA expression in PC3 cells untreated (CTR) or infected with scramble (shCTR) or NF-YA-targeting shRNA (shNF-YA). Tubulin was used as loading control. **D** Colony number of shCTR and shNF-YA cells cultured in anchorage-independent growth conditions. Data represent mean ± SEM (unpaired t-test: ***p < 0.001, *n* = 4). **E** Percentage of cell migration of shCTR and shNF-YA cells measured by transwell assay. Data represent mean ± SEM (unpaired t-test: *p < 0.05, *n* = 3). **F** Percentage of cell invasion of shCTR and shNF-YA cells measured by transwell assay. Data represent mean ± SEM (unpaired t-test: **p < 0.01, *n* = 2). **G** Representation of tumor incidence after 5 weeks from s.c. injection of shCTR and shNF-YA PC3 cells into SCID Hairless Outbred (SHO®) mice. **H** (Left panel) Volumes (mm^3^) of shCTR and shNF-YA xenografted tumors at the indicated time points. Data represent mean ± SEM (two-way ANOVA with Holm-Sidak’s test: **p < 0.01, ***p < 0.001, ****p < 0.0001, *n* = 7). (Right panel) Tumor weight of xenografted tumors isolated after 5 weeks from cell inoculation. Data represent mean ± SEM (unpaired t-test: **p < 0.01)

We then decided to investigate whether the expression of NF-YA isoforms is altered in cancer epithelial prostate cells compared to normal ones. Western blot analysis of total cellular extracts from different prostate cell lines clearly showed that healthy (N1) and benign hyperplasia (BPH) cells (C10, C17) almost exclusively express the longer NF-YA variant (NF-YAl) (Fig. 2A) [23]. NF-YAs was well detectable in SV40-immortalized PNT1A normal luminal cells, HPV-immortalized RWPE-1 normal basal cells and highly expressed in both androgen-unresponsive (PC3 and DU145) and androgen-responsive (LNCaP) cancer cells derived from different PCa metastastic sites [34]. The NF-YAs/NF-YAl ratio measured by qRT-PCRs perfectly matched with protein expression of NF-YA isoforms (Fig. 2B), indicating that gene transcription regulates NF-YAs and NF-YAl cellular levels.

The importance of NF-YA in cancer-associated cellular processes has been highlighted in various cellular contexts and NF-YA knock-down triggers cell cycle arrest and apoptotic cell death [9, 16–18, 35–37]. Therefore, we decided to analyze the effect of NF-YA knock-down in PC3 prostate cancer cells derived from a bone metastasis that show features of highly aggressive small cell neuroendocrine carcinoma (SCNC), an aggressive histological subtype of CRPC (metastatic castration-resistant prostate cancer). Lentiviral delivery of shRNAs led to high efficiency of gene inactivation (Fig. 2C) and time-course MTT analysis showed a slight but significant reduction in cellular proliferation of shNF-YA cells compared to shCTR ones (Suppl. Fig. 1A). We then evaluated the effect of NF-YA abrogation on cancer-related cellular processes, such as clonal growth, migration and invasion abilities. The soft agar colony formation assay highlighted a clear impairment in anchorage-independent growth of NF-YA-inactivated cells when compared to control cells (Fig. 2D). Additionally, NF-YA loss induced severe defects in migration and invasion abilities of PC3 cancer cells, as demonstrated by measurements in Transwell two-chamber system with FBS as chemoattractant (Fig. 2E and F).

Finally, we verified whether NF-YA depletion affects the tumorigenic potential of PC3 cells in vivo*.* Scramble and shNF-YA-infected PC3 cells were sub-cutaneously (s.c.) injected into immune-compromised mice and xenograft tumor growth was monitored for 5 consecutive weeks. NF-YA loss severely impaired tumor incidence and growth of engrafted tumors (Fig. 2G, H and Suppl. Fig. 1B).

These results demonstrated the important role of NF-YA expression in PCa-associated phenotype.

### NF-YAs overexpression enhances cell proliferation of PCa cells within three-dimensional multicellular spheroids

**Fig. 3:**
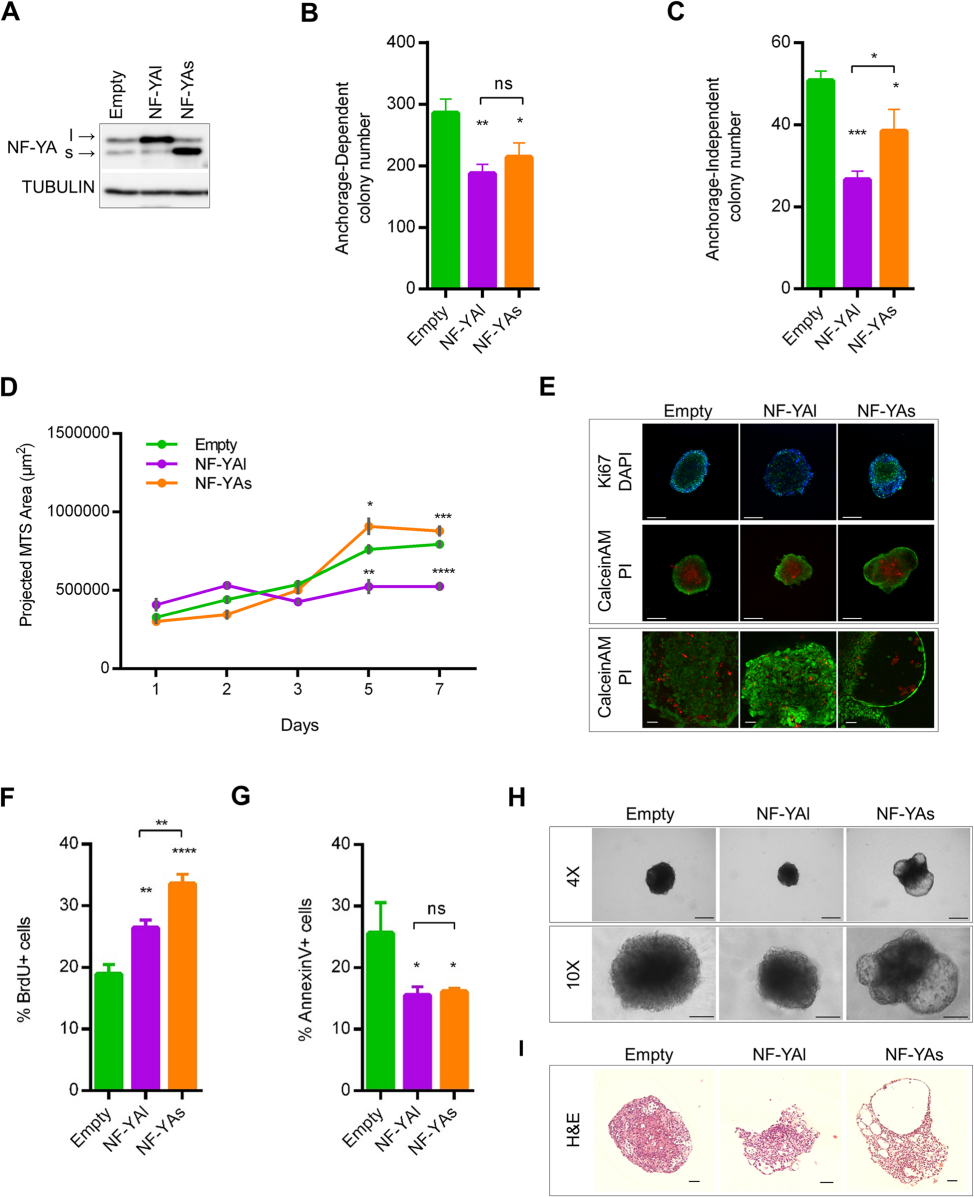
Effects of stable overexpression of NF-YA isoforms in tumor cells. **A** Western blot analysis of total extracts from PC3 cells stably infected with Empty, NF-YAl and NF-YAs lentiviral particles. Tubulin was used as loading control. **B, C** Colony number of Empty, NF-YAl and NF-YAs stable cell lines in anchorage-dependent and anchorage-independent conditions, respectively. Data represent mean ± SEM (one-way ANOVA with Fisher’s LSD test: *p < 0.05, **p < 0.01, ***p < 0.001, ns, not significant, *n* = 6). **D** Size of Empty, NF-YAl and NF-YAs MTSs calculated as projected area at the indicated time points. Data represent mean ± SEM (two-way ANOVA with Holm-Sidak’s test: *p < 0.05, **p < 0.01, ***p < 0.001, ****p < 0.0001, n between 8 and 68 spheroids from 8 independent experiments). **E** Representative immunofluorescence images of Ki67/Dapi and Calcein-AM/PI staining performed on Empty, NF-YAl and NF-YAs MTSs. Scale bar = 500 μm. The lower panel shows details of Calcein-AM/PI staining by confocal microscopy. Scale bar = 50 μm. **F** Percentage of BrdU-positive cells following incubation of MTSs with 20 μM BrdU for 16 h. Data represent mean ± SEM (one-way ANOVA with Fisher’s LSD test: **p < 0.01, ****p < 0.0001, *n* = 4). **G** Percentage of AnnexinV-positive cells identified by cytofluorimetric analysis of MTSs. Data represent mean ± SEM (one-way ANOVA with Fisher’s LSD test: *p < 0.05, ns, not significant, *n* = 3). **H** Optical microscopy images representative of Empty, NF-YAs and NF-YAl MTSs morphology. Scale bar 4X = 500 μm, scale bar 10X = 250 μm (I) Representative images of H&E stained sections of MTSs. Scale bar = 100 μm

To understand whether NF-YAs, compared to NF-YAl, has unique properties in conferring aggressiveness to PCa, we generated PC3 (Fig. 3A), LNCaP and DU145 (Suppl. Fig. 2A, E) cell lines stably overexpressing NF-YA isoforms through transduction of NF-YAs- and NF-YAl- lentiviral particles. A decrease in clonal expansion was observed in NF-YA-overexpressing cells when cultured in anchorage-dependent (Fig. 3B and Suppl. Fig. 2B) and anchorage-independent conditions (Fig. 3C and Suppl. Fig. 2C).

To better understand the differences induced by NF-YAs and NF-YAl in PCa cells, we took advantage of in vitro 3D models that closely reproduce relevant tumor conditions for complex cell–cell and cell–matrix interactions. Multicellular tumor spheroids (MTSs) were grown in scaffold-based or -free conditions, depending on the ability of PCa cell lines to spontaneously form spheroids in anchorage-independent circumstances. NF-YAs overexpression increased spheroids size of PC3 (Fig. 3D), LNCaP and DU145 (Suppl. Fig. 2D, F).

We assessed cell proliferation and viability in MTSs through Ki-67 and Calcein-AM/Propidium Iodide (live/dead) stainings: all MTSs showed an external proliferating zone and an internal necrotic area (Fig. 3E, upper and middle panels). Cytofluorimetric analysis of BrdU-stained-cells was then performed: a higher percentage of BrdU-positive cells was detected in NF-YA-overexpressing MTSs, in particular in NF-YAs-spheroids (from 18.9% in Empty to 26.4 and 33.6% in NF-YAl and NF-YAs, respectively) (Fig. 3F), hinting at increased proliferation rate induced by NF-YAs overexpression. Significant decrease of apoptotic/necrotic Annexin V-positive cells was observed in both NF-YAl- and NF-YAs-MTSs (Fig. 3G). The increase in activated AKT, detected by phosphorylation at Ser473 in MTS extracts, was consistent with a major activity in cell proliferation for NF-YAs with respect to NF-YAl (Suppl. Fig. 2G).

Interestingly, morphological analysis of Calcein-AM/Propidium Iodide-stained MTSs by confocal microscopy (Fig. 3E, lower panel), optical microscopy and hematoxylin and eosin staining (H&E) (Fig.3H, I) showed dramatic phenotypic alterations with budding and ring-shaped structures in NF-YAs-MTSs.

Taken together, these findings demonstrate that NF-YAs enhances cell proliferation and triggers unique cell-ECM interactions in three-dimensional culture conditions recapitulating complex interactions.

### NF-YAs expression is required for PCa cell viability

**Fig. 4:**
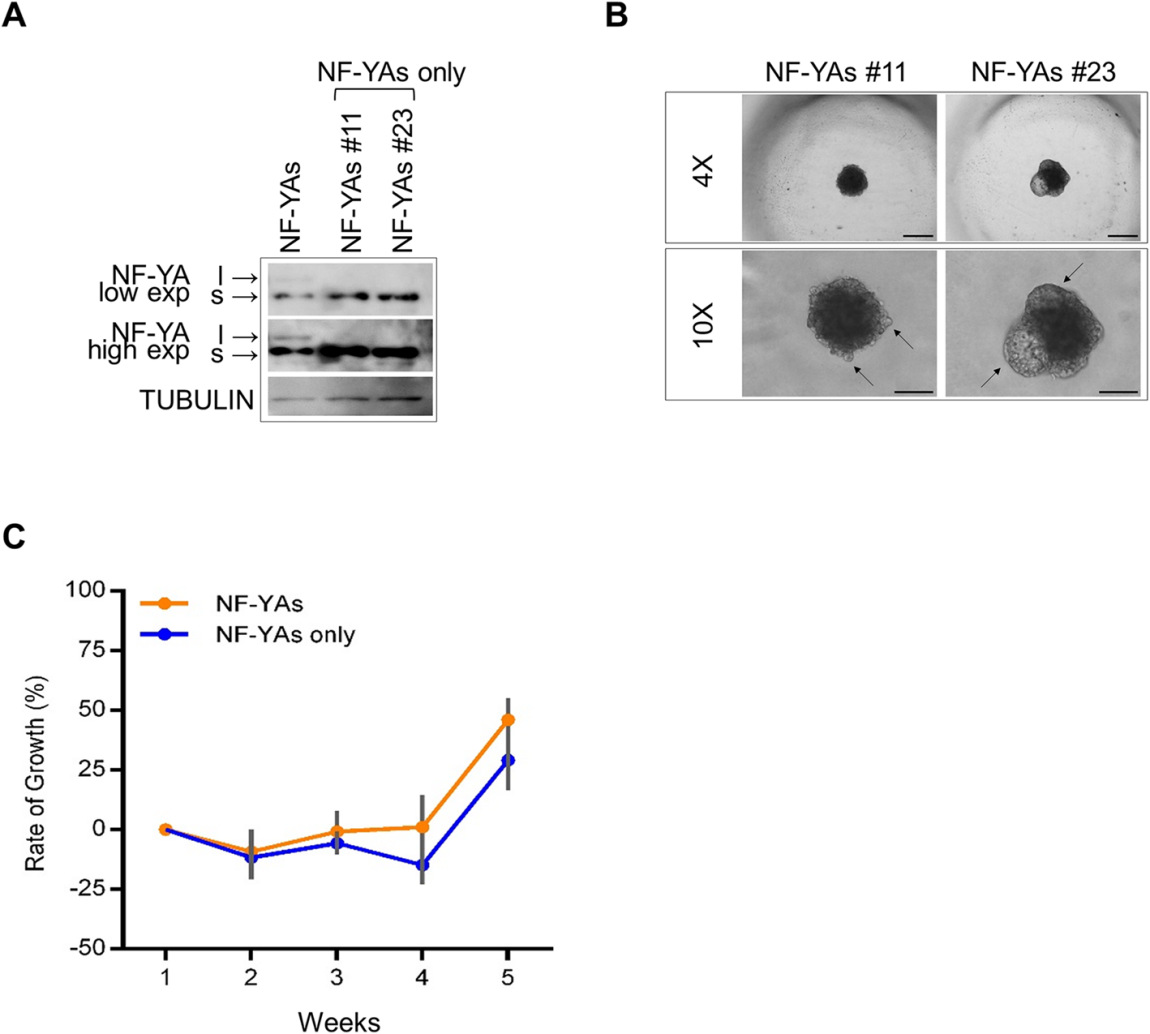
Effect of CRISPR-Cas9-mediated knock out of endogenous *NF-YA* in NF-YAs-overexpressing PC3 cells. **A** Western blot analysis of NF-YA isoforms in total cellular extracts from NF-YAs and NF-YAs-only (CRISPR-Cas9 edited clone #11 and #23) PC3 cells. Tubulin was used as loading control. **B** Optical microscopy images representative of the morphology of two different NF-YAs clones cultured as MTSs. The arrows indicate structures budding from MTS core. Scale bar 4X = 500 μm, scale bar 10X = 250 μm. **C** Rate of growth (%) of xenograft tumors measured for 5 consecutive weeks from s.c. inoculation of NF-YAs and NF-YAs-only edited PC3 cells into SCID Hairless Outbred (SHO®) mice. Data represent mean ± SEM (Two-way ANOVA with Holm-Sidak’s test *n* = 4)

Although the levels of endogenous human NF-YAs or NF-YAl could be considered negligible compared to the overexpressed murine isoform (human-mouse protein identity = 99%) (Fig. 3A), we knocked down the expression of endogenous *NF-YA* gene to definitely ascribe phenotypic alterations to isoform-specific activities. We designed a sgRNA to guide *Streptococcus pyogenes* Cas9 (SpCas9) to a 5′-TGG-3′ PAM sequence present on the reverse complementary strand of human NF-YA exon 2 (Suppl. Fig. 3A). NF-YAs and NF-YAl PC3 cells were transfected with control or effector plasmids for the expression of CRISPR/Cas9 components and GFP reporter gene, and analyzed for frequency of editing 2 days post-transfection by TIDE analysis. CRISPR-treated PC3 cells showed about 38% transfection efficiency in both NF-YAs and NF-YAl cells. We observed up to 12.9% editing on the human *NF-YA* gene (Suppl. Fig. 3B), without detectable indel frequency on the overexpressed murine isoforms (Suppl. Fig. 3C). To enrich for edited cells, we sorted GFP+ transfected cells by flow cytometry and isolated monoclonal cell populations. The number of CRISPR-edited clones for NF-YAl cells was dramatically reduced compared to NF-YAs ones (6% vs 41%, respectively, in 3 experiments). TIDE analysis of NF-YAl edited clones did not identify biallelic *NF-YA* deletion, while 82% of NF-YAs edited clones harbored biallelic deletion. These results indicate that cells expressing uniquely NF-YAl (NF-YAl-only) cannot survive, differently from the cells expressing NF-YAs-only. We thus selected two NF-YAs-only clones for further analyses (Fig. 4A and Suppl. Fig. 3D). The clones showed morphological characteristics similar to NF-YAs MTSs (Fig. 4B). Additionally, NF-YAs-only cells formed tumors in vivo with 100% incidence and a tumor growth similar to NF-YAs xenograft tumors (Fig. 4C).

These results suggest a unique role for NF-YAs in PCa cell viability.

### The transcriptional profiles activated by NF-YAs and NF-YAl differently overlap with human localized or metastatic PCa signatures

**Fig. 5:**
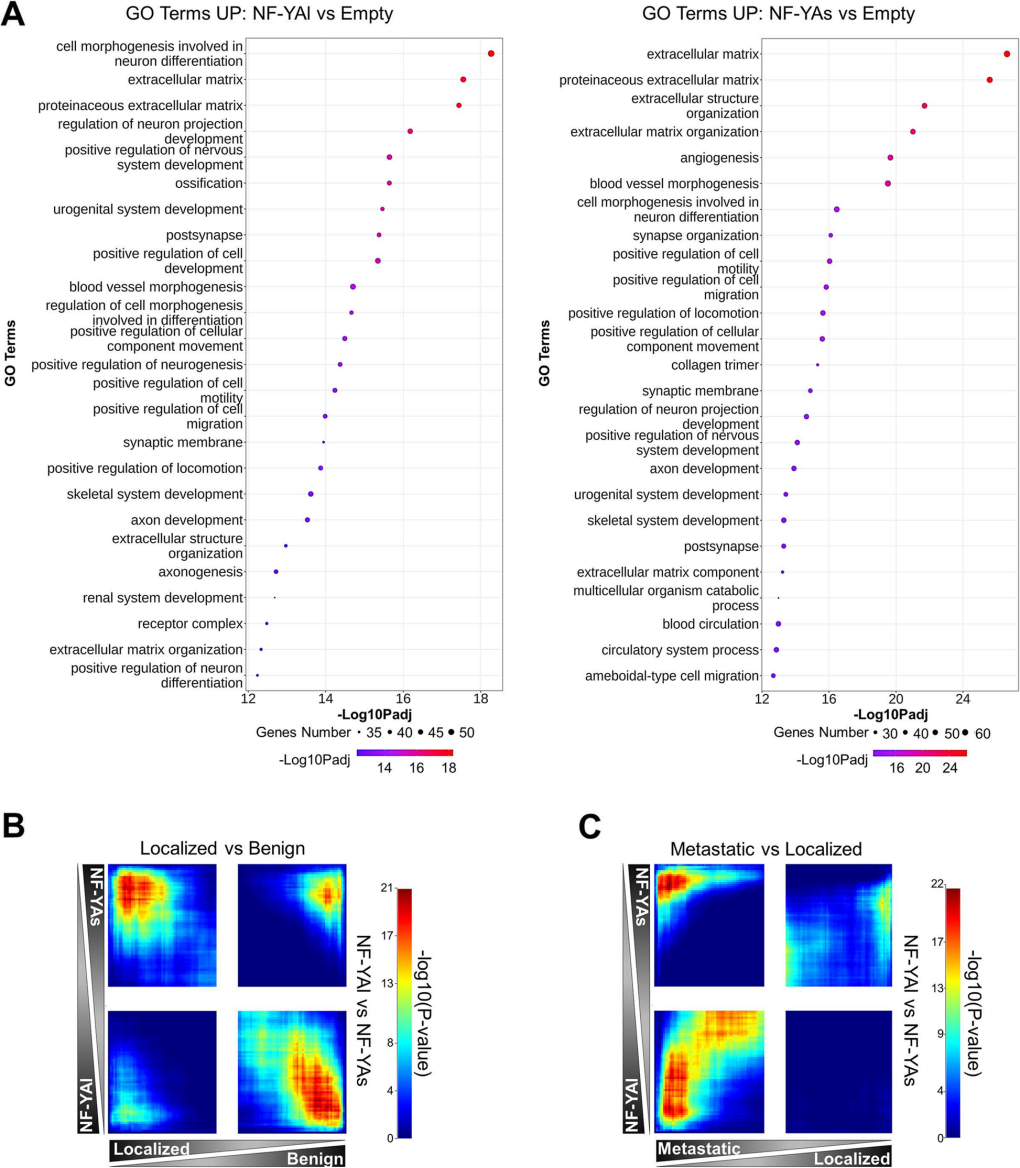
Expression profiles of NF-YAs and NF-YAl overexpressing MTSs. **A** Top 25 enriched GO terms of up regulated genes in NF-YAl (left panel) and NF-YAs (right panel) overexpressing PC3 MTSs versus Empty control ones. The size of each circle represents the number of genes included in each GO term and the color of the circle indicates the adjusted *p* value. **B** Comparison of gene expression signatures between NF-YAl vs NF-YAs MTSs and Localized vs Benign prostate tissues [38] by RRHO analysis. **C** Comparison of gene expression signatures between NF-YAl vs NF-YAs MTSs and Metastatic vs Localized prostate tissues [38] by RRHO analysis

To shed light on the transcriptional effects induced by NF-YA isoforms, leading to different tumor phenotypes, we performed RNA-seq profilings of transduced cells cultured as MTSs. Compared to Empty-cells, 872 and 620 differentially expressed genes (DEGs) were retrieved in NF-YAl cells, and 962 and 686 genes were found in NF-YAs cells as up- and down-regulated, respectively. The analysis of Gene Ontology enrichment (GO< 500 and |Log2FC| > 1) (Fig. 5A and Suppl. Fig. 4) showed that GO Terms up-regulated in NF-YAl cells are associated to neuron differentiation and nervous system, urogenital development, cell morphogenesis, extracellular matrix (ECM) and positive regulation of cell motility/migration. The overexpression of NF-YAs induced the up-regulation of gene categories related to ECM organization, angiogenesis and blood vessels morphogenesis, cell motility/migration and locomotion. Since some terms were common between NF-YAl and NF-YAs transcriptional profiles, we performed a selective analysis of NF-YAl vs Empty unique DEGs by filtering out genes with |Log2FC| > 0.5 in NF-YAs vs Empty, and vice versa for NF-YAs vs Empty unique DEGs, discarding genes with |Log2FC| > 0.5 in NF-YAl vs Empty (Suppl. Fig. 5). Categories associated to cell differentiation, in particular neuron differentiation, were still retrieved among NF-YAl up-regulated GO terms (Suppl. Fig. 5A, left panel), while NF-YAs confirmed mainly ECM component and organization as increased gene products (Suppl. Fig. 5B, left panel).

The ECM signature clearly hints at a role for NF-YAs in modulating the interaction between epithelial cells and the environment, which is consistent with the budding ring-shaped structures previously described (Fig. 3H, I) but also suggestive of an invasive phenotype. In order to identify whether the NF-YA splicing signature could correlate with PCa aggressiveness and metastatic potential, we surveyed genes highly expressed in localized vs benign and metastatic vs localized human tumors data sets [38] and checked for concordant and discordant gene expression signatures using an updated rank-rank hypergeometric overlap (RRHO) approach [39]. RRHO heatmaps showed anti-correlation between genes overexpressed in NF-YAl vs NF-YAs and those upregulated in localized vs benign samples (Fig. 5B). An extensive biologically relevant overlap was observed between the signature of metastatic vs localized tumors and genes highly expressed in both NF-YAl and NF-YAs (Fig. 5C).

These results suggest that NF-YAs expression characterizes localized tumors, while both NF-YAs and NF-YAl could be involved in the metastatization process.

### Effect of NF-YAs and NF-YAl overexpression on PCa malignant features in vivo

**Fig. 6:**
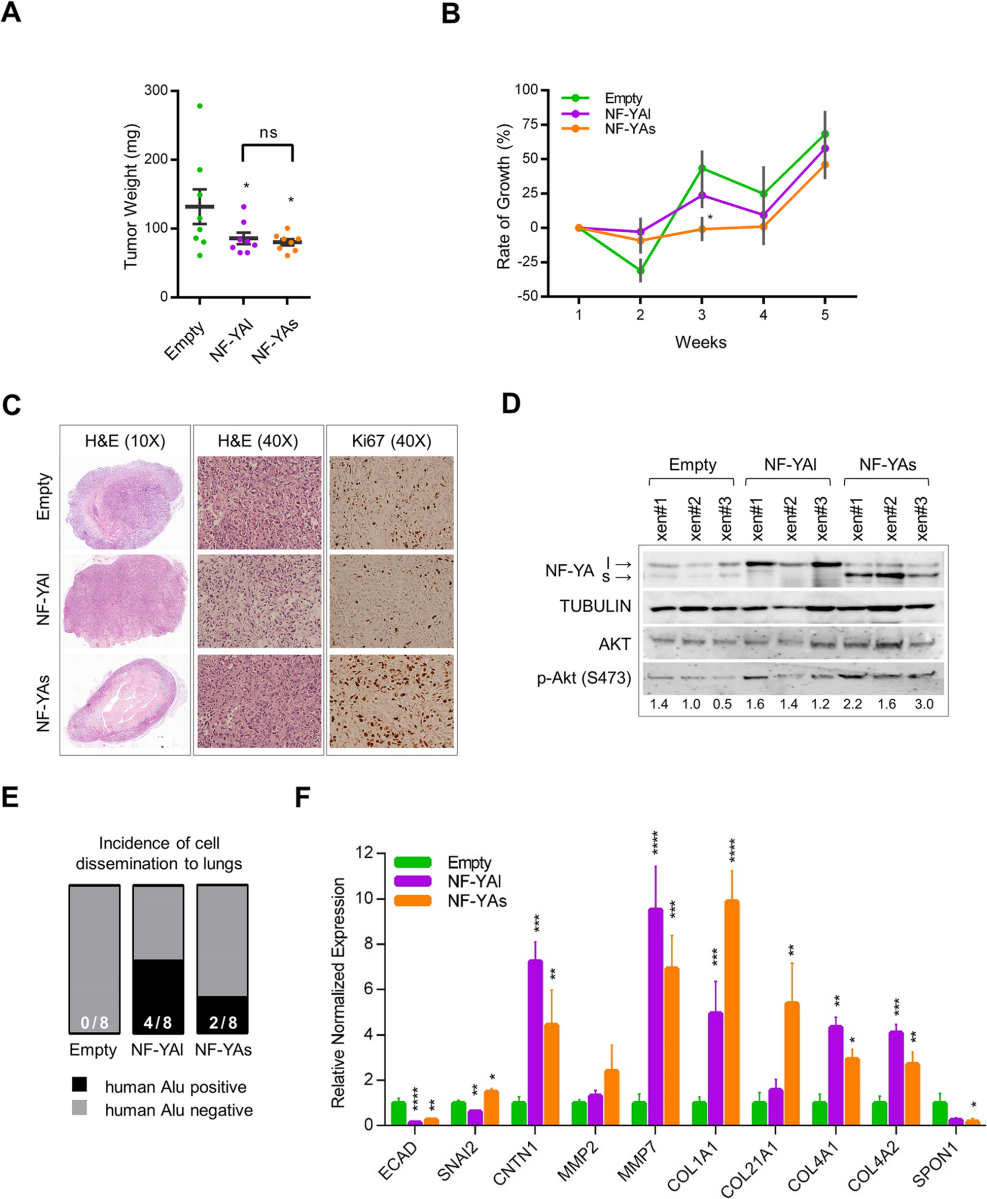
Effect of NF-YAs and NF-YAl overexpression on tumor growth and cell dissemination in vivo. **A** Weight (mg) of xenograft tumors at 5 weeks from s.c. inoculation of Empty, NF-YAl and NF-YAs PC3 cells into SCID Hairless Outbred (SHO®) mice. Data represent mean ± SEM (one-way ANOVA with Fisher’s LSD test: *p < 0.05, ns, not significant, *n* = 8). **B** Rate of growth (%) of Empty, NF-YAs and NF-YAl xenograft tumors at the indicated time points following s.c. inoculation of transduced PC3 cells. Data represent mean ± SEM (two-way ANOVA with Holm-Sidak’s test: *p < 0.05, *n* = 8) **C** Representative H&E-stained FFPE sections of 5 weeks xenograft tumors at low (left panel) and high (middle panel) magnifications. Immunohistochemical detection of Ki67 in xenograft tumors (right panel). **D** Western blot of total extracts from PC3 tumor xenograft with the indicated antibodies. Tubulin has been used as loading control. Quantification of band intensities was performed with ImageJ software and relative phospho-AKT(Ser473) levels are indicated, after normalization to Tubulin and AKT expression (**E**) Representation of the incidence of spontaneous cell dissemination to lung tissue in mice after 5 weeks from s.c. injection of Empty, NF-YAl, NF-YAs PC3 cells. Cell dissemination has been identified by the detection of human genomic DNA through Alu-qPCR in mouse lung tissues. **F** RT-qPCR analysis of the indicated transcripts in xenograft tumors harvested following 5 weeks from s.c. injection. Rps20 and b-Actin were used as reference genes and normalized mRNA levels are reported as fold change vs Empty biological group, arbitrarily set at 1. Data represent mean ± SEM (one-way ANOVA with Tukey’s test: *p < 0.05, **p < 0.01, ***p < 0.001, ****p < 0.0001, *n* = 5)

We then used NF-YA transduced cells for mouse xenograft studies to investigate whether the above described properties were obvious also in vivo. Equal number of exponentially growing PC3 cells were injected s.c. into SCID Hairless mice and tumor volumes were determined (Suppl. Fig. 6A, B). Both NF-YAs and NF-YAl overexpression significantly reduced tumor weights at 5 weeks post-injection (Fig. 6A). Taking into consideration that very few injected PC3 cells initiate the tumor [40], reduced tumor size could be the consequence of decreased clonal ability observed in NF-YA-transduced cells (Fig. 3B, C). Indeed, the analysis of growth ability of engrafted cells, evaluated as rate of growth (%), showed similar kinetics in NF-YA-overexpressing tumors compared to Empty-ones from week 4 (Fig. 6B). Moreover, we observed a lag in the growth of NF-YAs tumors before the exponential phase, which could be the consequence of the complex cell-ECM interaction previously described. We decided to process xenograft tumors for histopathological analysis with routine H&E staining and Ki-67 immunohistochemistry (Fig. 6C), used in clinical practice to assess tumor aggressiveness. Empty tumors showed moderate tumor cellularity, predominantly composed by epithelioid round cells with respect to spindle cells, and thin bands of hyaline collagenous-like matrix. Ki-67 label index ranged from 15 to 24%. NF-YAl overexpression formed tumor tissue with moderate to mild cellularity. Scattered round cells and occasionally spindle cells with inconspicuous nuclei were observed in the stromal background. Ki-67 label index ranged from 5 to 17%. Differently, NF-YAs tumors showed highly cellular tissue composed by epithelioid elements with irregular large nuclei and evident nucleoli close to each other’s with no stroma in the peripheral layer, where Ki-67 label index ranged from 28 to 67%, corroborating a high proliferative activity. Next to the outer proliferative rim, a necrotic core was evident, which can result from rapid tumor cell growth and correlates with adverse outcomes in numerous solid tumors [41–43]. We further analyzed other markers related to tumor progression by western blot analysis of total extracts from xenografts (Fig. 6D). An increase in AKT and its phosphorylated form, both correlated with aggressive cancer [44, 45], was evident in NF-YA overexpressing tumors, particularly in NF-YAs ones.

Despite subcutaneous PC3-xenografts should not yield metastatic lesions within 5 weeks [46], qPCR-mediated amplification of human specific-Alu sequences was used to detect possible disseminated human tumor cells within lungs from tumor-bearing mice. As expected, no amplification of human genomic DNA was obtained from lungs of mice after s.c. injection of control Empty PC3 cells. Differently, we observed Alu-positive cells in lungs of 2 and 4 out of 8 mice after injection with NF-YAs and NF-YAl cells, respectively (Fig. 6E).

RNA extraction from xenograft tumors followed by RT-qPCR on selected genes that participate to PCa metastatization showed a significant transcriptional modulation in both NF-YAs and NF-YAl samples compared to Empty tumors (Fig. 6F).

These results suggest that NF-YA overexpression does not favor tumor formation, rather confers aggressive hallmarks to established tumors with NF-YAs enhancing proliferation and NF-YAl favoring cell dissemination.

### NF-YAs and NF-YAl overexpression leads to different invasive behaviors

**Fig. 7:**
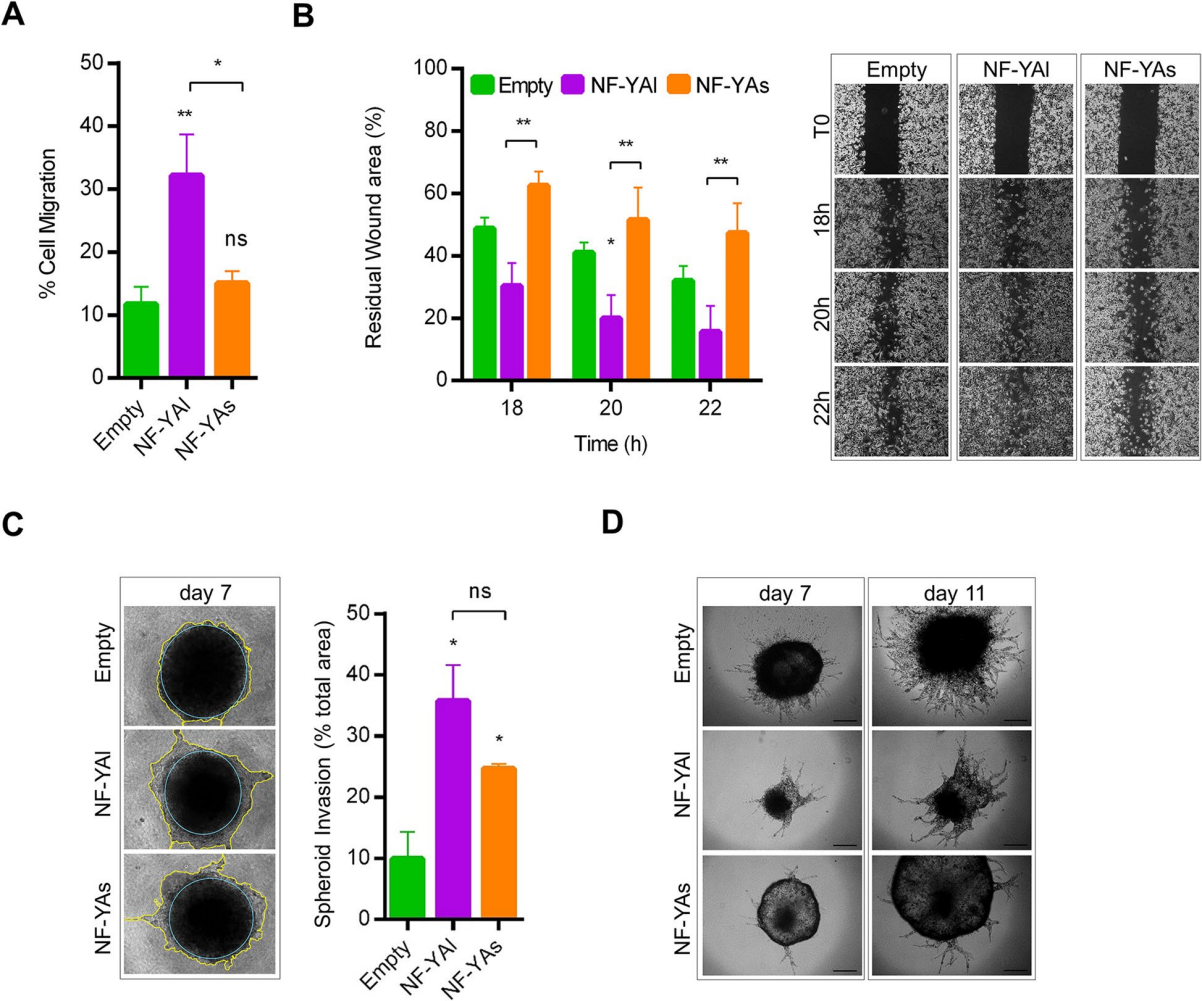
Activity of NF-YAs and NF-YAl overexpression in cell migration and 3D invasion. **A** Percentage of cell migration of Empty, NF-YAl and NF-YAs cells measured by transwell assay. Data represent mean ± SEM (one-way ANOVA with Fisher’s LSD test: *p < 0.05, **p < 0.01, ns, not significant, *n* = 4). **B** Confluent cells were seeded into Ibidi wound-healing chambers and images were acquired at 0, 18, 20 and 22 h after the culture chambers were removed. (Left panel) Quantification of migration is shown as residual wound area compared to the initial gap, arbitrarily set at 100%. Data represent mean ± SEM (two-way ANOVA with Fisher’s LSD test: *p < 0.05, **p < 0.01 *n* = 6). (Right panel) Representative images of cell migration in wound-healing assay. **C** (Left panel) Representative phase contrast microscopy images of Empty, NF-YAl and NF-YAs MTSs. Spheroid invasion of MTSs included for 7 days in 1 mg/ml growth factors-enriched Matrigel. (Right panel) The histogram represents the percentage of invasion area vs total area of MTSs. Data represent mean ± SEM (unpaired t-test: *p < 0.05, ns, not significant, *n* = 3). **D** Phase contrast microscopy images representative of the invasive behaviour of MTSs when embedded into 5 mg/ml growth factors-reduced Matrigel surrounded by high-serum culture medium as chemoattractant for 7 and 11 days (*n* = 4). Scale bar = 500 μm

The unexpected higher disseminating ability of NF-YAl vs NF-YAs cells in xenografts hinted at a more important role for NF-YAl in the multi-step metastatic process compared to NF-YAs cells. Therefore, we performed cell migration and invasion in vitro assays to compare NF-YAl and NF-YAs pro-metastatic activities. Boyden chamber assays showed increased ability of single cell motility toward a chemo-attractant gradient in NF-YAl-cells (Fig. 7A). Besides, in wound healing 2D assay, NF-YAl cells closed the gap faster than NF-YAs cells (Fig. 7B).

We then analyzed size and shape in 3D ECM spheroid invasion assays. PC3 aggregates were included in 1 mg/ml growth factors-enriched Matrigel, which allowed both proliferation and invasion. Optical microscopy showed that some cells leave the core of Matrigel-embedded MTSs, forming a more evident invasion zone in NF-YAs and NF-YAl compared to Empty spheroids (Fig. 7C). Additionally, we studied the invasive behavior of MTSs when embedded into 5 mg/ml growth factors-reduced Matrigel and surrounded by high-serum culture medium as chemo-attractant (Fig. 7D). The results showed that NF-YAl induces the formation of multicellular strands radiating from the periphery of the spheroid, with sunburst pattern similarly to Empty MTSs. Differently, NF-YAs MTSs have an expansive ring-like budding phenotype, as already observed in 3D growth condition (Fig. 3H).

Overall, both NF-YA isoforms could contribute to tumor invasion, but only NF-YAl increase enhances cell migration.

### NF-YAs expression predicts the clinical outcome of PCa patients

**Fig. 8:**
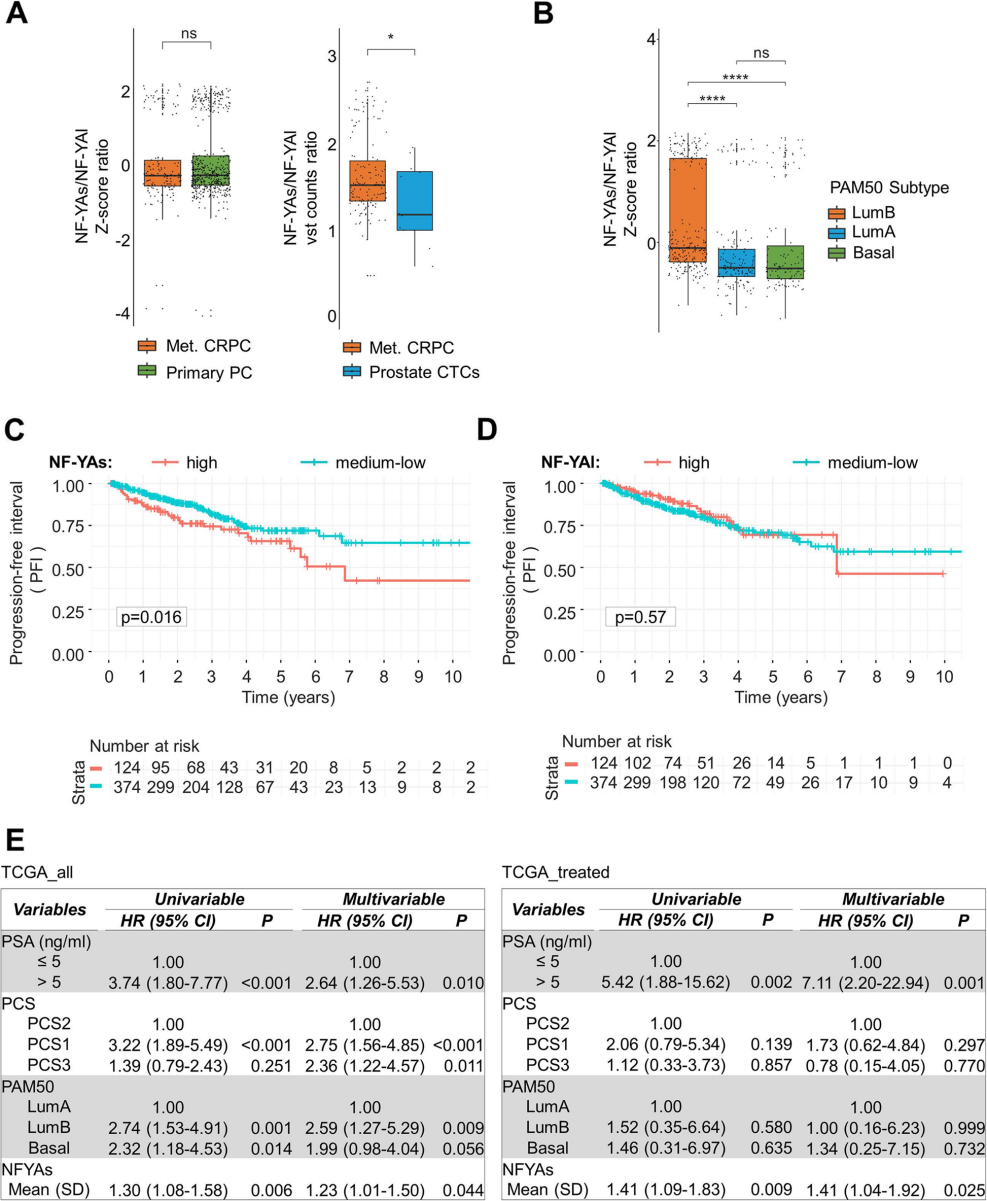
Lower NF-YAs/NF-YAl ratio distinguishes PCa CTCs from Met. CRPC and high NF-YAs predicts the clinical outcome of PCa patients. **A** Left panel: Ratio of NF-YAs/NF-YAl transcripts in Met. CRPC samples compared to TCGA primary adenocarcinomas. Box plots were obtained following log2 transformation of TPM and z-score normalization of processed datasets. Right panel: Ratio of NF-YA isoforms in Met. CRPC samples compared to PCa CTCs. Raw counts of both datasets were normalized with DESeq2 algorithm and the Variance Stabilizing Transformed (vst) is represented. **B** Log transformed TPM and z-score normalization of NF-YAs/NF-YAl ratio in PRAD samples, stratified according to PAM50 subtypes. Wilcoxon test: ****p < 0.0001, ns, not significant. **C, D** Kaplan-Meier analysis of progression-free interval (PFI) in TCGA PRAD patients stratified according to high and medium/low expression of NF-YAs and NF-YAl, respectively. *P* values for the log rank tests are indicated. **E** Univariable and multivariable hazard ratio (HR) analyses of different prognostic parameters and NF-YAs expression in PRAD patients. Left table includes all patients; right table includes a subset of patients who have received either radiotherapy or chemotherapy

These results prompted us to analyze the levels of NF-YAs and NF-YAl transcripts in patients affected by metastatic CRPCs [47] compared to primary adenocarcinomas. The ratio between NF-YAs/NF-YAl did not decrease in metastatic disease, independently from the metastatic site, ruling out that NF-YAl increase could induce metastatic colonization of other tissues (Fig. 8A, left panel and Suppl. Fig. 6C). Since our in vitro and in vivo results clearly indicated a possible role of NF-YAl in cell migration, we decided to explore RNA-seq data of CTCs (circulating tumor cells). Although data were available only from few patients [48], the results showed a significant reduction in NF-YAs/NF-YAl ratio compared to metastatic samples (Fig. 8A, right panel), supporting our hypothesis that increased NF-YAl could enhance migrating abilities of PCa cells.

To investigate a possible application of NF-YA splicing signature in stratification of PCa patients, we used a transcriptome-based classification method associated with clinical behavior [5, 49–51]. We applied the PAM50 algorithm to identify whether the NF-YA splicing signature is altered in previously characterized luminal- and basal-like PCa subtypes that show differences in clinical outcomes and treatment response [5]. In opposition to NF-YAl, NF-YAs is particularly high in LumB subtype, which has the poorest prognosis even with low Gleason sum score (GS < 7) and is characterized by a proliferative signature [5, 6] (Fig. 8B).

We then decided to determine whether the expression of NF-YA splice variants can be used as a prognostic marker for PCa. In PRAD TCGA studies survival analyses were not reported, therefore the use of progression-free interval (PFI) from prostatectomy has been recommended [52]. Stratification on the basis of high or medium/low NF-YAs expression highlighted a significant poorest clinical prognosis of PCa patients characterized by high NF-YAs expression (Fig. 8C). Despite not significant, lower PFI was observed in medium/low NF-YAl patients up to 4 years (Fig. 8D). Next, we used the Cox proportional-hazards model for investigating the association between the survival time of patients, represented by hazard ratio (HR), and NF-YAs expression together with PSA (ng/ml), PCS and PAM50 variables. Higher NF-YAs levels significantly increase HR both in TCGA cohort that includes all PCa patients (Fig. 8E, left panel) and in the sub-cohort consisting of patients treated with pharmacological or radiation therapy (Fig. 8E, right panel).

These results further corroborate that high level of NF-YAs transcript is a significant independent prognostic factor associated with poor clinical outcome in PCa patients.

## Discussion

PCa disease is characterized by extreme clinical heterogeneity and unpredicted therapeutic response of patients. The transcriptional profile of PCa is different from the majority of tumors, being an indolent and slow-proliferating cancer in which cell growth and cell cycle gene categories are not retrieved among up-regulated terms. Despite this, large-scale analyses identified NF-Y among TFs involved in molecular networks inducing the progression from benign epithelium to both localized and hormone-refractory metastatic PCa [7]. Moreover, NF-Y has been listed among TF coordinated groups (TFCGs) that characterize the differential transcriptional signature in tumors of patients treated with ADT, which is enriched in genes that drive metastasis [50].

Data analysis from TCGA database highlights the increase in total NF-YA gene transcription in PCa compared to healthy tissues, in particular in high GS samples. This increase is associated to the up-regulation of the NF-YAs isoform, while NF-YAl transcript decreases. Of particular relevance is the demonstration that changes in the expression of NF-YA isoforms are associated to key clinical and molecular features of aggressive PCa. The ratio between NF-YAs and NF-YAl increases in higher GS adenocarcinomas and in luminal B/PCS1 tumor subtype, which includes tumors with the poorest outcome with GS ≥ 8 but also with GS ≤ 7 associated to metastatic progression [6] (Figs. 1 and 8).

In vivo analysis of the effects of NF-YA overexpression on tumor growth led to unexpected results: although increased NF-YAs levels should enhance tumor progression, NF-YAs xenograft tumors showed reduced volume compared to control tumors. A similar behavior was observed for NF-YAl xenografts. Mass spectrometry technique has recently demonstrated that very few injected cells initiate the tumor regardless of the quantity of delivered cells, which showed more than 95% cell death [40]. Since s.c. tumors are formed by a very small population of tumor initiating cells, the reduced clonogenic ability induced by both NF-YAs and NF-YAl overexpression could affect tumor engrafment (Fig. 6).

Because of ethical issues, we could not follow tumor growth beyond week 5. Therefore, we performed histological analysis of xenograft tumors to better characterize the effect of NF-YA overexpression on tumor growth. NF-YAs tumors showed a highly Ki67+ proliferative rim and an internal necrotic core. The presence of necrosis within tumors is controversial: on one hand necrosis supports shrinkage of tumor mass and consequently appears as a favorable characteristic, on the other hand necrosis promotes tumor progression and aggressiveness and is considered as a negative prognostic factor (for a review see [41]). Western blot analysis on xenograft total extracts identified an increase in phospho-AKT particularly in NF-YAs tumors, suggesting once again a more aggressive phenotype.

MTSs, models for self-assembled cell aggregates, came in support to better elucidate whether NF-YAs overexpression can sustain tumor aggressiveness, as suggested by TCGA transcriptional data from PCa patients. A significant increase in BrdU+ cells demonstrates the enhanced proliferative ability conferred by NF-YAs to tumor cells compared to both Empty and NF-YAl cells, accompanied by reduced cell death determined by Annexin V staining. Surprisingly, no categories associated to cell proliferation or cell cycle progression are observed in highly proliferative NF-YAs tumors, although these terms are usually described as up-regulated by NF-YA overexpression [15–17, 20]. The main up-regulated GO terms are associated to ECM organization, which could account for evident changes in MTSs morphology. Tumor progression demands continuous interactions between ECM and tumor cells, which secrete matrix-metalloproteinases (MMPs), fibronectin and collagens that interfere with cell-cell adhesion and cell polarity [53]. Consistently, many genes belonging to these categories transcriptionally increase in NF-YAs-xenograft tumors. Despite this, cell migration does not seem to characterize NF-YAs cells but NF-YAl ones, at least when analyzed by 2D wound healing and transwell-migration assays (Fig. 7). When cultured in 3D, NF-YAl cells show sprouting cells associated to reduced MTS dimension, while NF-YAs cells form bigger MTSs characterized by ring-like structures from which some cells can sprout, in particular at later time points. These results would suggest that NF-YAs has mainly a pro-proliferative activity, but once cell–cell and cell–matrix interactions occur, it could confer invasive properties too.

Taking into consideration that high NF-YAs/NF-YAl ratio characterizes both primary tumors and metastatic ones, we hypothesized that high NF-YAs is a condition necessary to allow tumor cell expansion locally as well as in distant metastasis, while NF-YAl could be required for short-time specific invasive behaviors, such as migrating abilities. Indeed, the metastatic process requires three consecutive steps: firstly, neoplastic cells break down the basement membrane of tumor blood vessels, allowing stroma invasion and intravasation. Afterwards, the cells have to survive through the circulation and they finally extravasate in a distant organ, where they adapt and start to proliferate [54]. The profile of circulating tumor cells (CTCs) from CRPC patients supported our hypothesis (Fig. 8). Although very few datasets from PCa CTCs are available and the majority of these data do not satisfy the criteria to allow the analysis of alternatively spliced transcripts [55–57], we were able to obtain preliminary results from a little cohort of patients [48]. The significant decrease in NF-YAs/NF-YAl ratio in CTCs compared to both primary and metastatic PCa, corroborate that higher NF-YAl expression can confer cell migration ability even in PCa patients.

PFI and regression analyses highlight the clinical significance of our studies. In particular, we propose the analysis of NF-YAs levels as a new molecular strategy for risk assessment in PCa patients. High NF-YAs expression is associated with PCa outcome, independently of other clinical variables. Optimal management of PCa is majorly associated to the diagnostic process rather than the selection of appropriate active treatment. While Gleason score, TNM staging and pre-treatment PSA values provide key prognostic information on the expected behaviour of the tumor, they don’t always allow to discriminate biologically aggressive tumors (for a review see [58]).

**Fig. 9:**
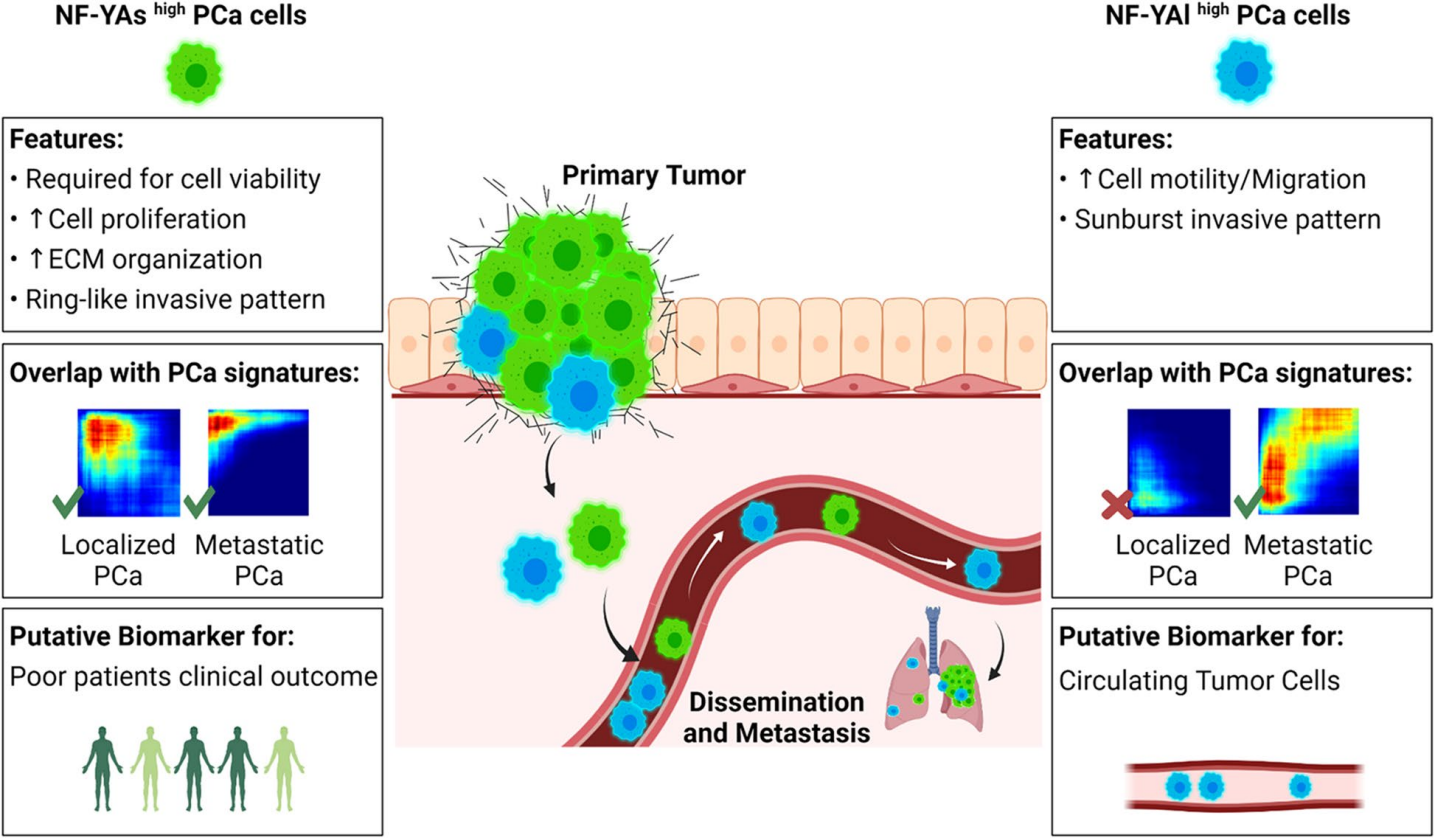
Schematic representation of biological properties of PCa cells overexpressing different NF-YA isoforms. Localized and metastatic PCa tumors are mainly composed by cells expressing high levels of NF-YAs (NF-YAs^high^), which is fundamental for their survival and enhances proliferation and interaction with ECM. Both NF-YAs^high^ and NF-YAl^high^ cells are able to invade ECM with different patterns and form distant lung metastases. NF-YAl^high^ cells have increased migration ability, consistently with higher NF-YAl expression in PCa CTCs. Stratification of patients based on NF-YAs^high^ signature predicts poor clinical outcome. Created with BioRender.com (Biorender, RRID:SCR_018361)

In this scenario, NF-YAs can represent a new biomarker for stratification of PCa patients (Fig. 9). Further studies will allow to investigate whether NF-YA splicing signature can improve active surveillance in low-risk PCa or properly classify aggressive cancers erroneously defined as “intermediate” risk on the basis of tumor biopsy, thus avoiding both over- or under-treatment. Finally, a deepen analysis of NF-YA splicing in CTCs will give new insights on the role of NF-Y in conferring ability to the cells to survive in the peripheral blood, resistance to anoikis, escape of immune surveillance and resistance to chemotherapy.

## Conclusions

Regulation of the splicing process of the NF-YA subunit of the transcription factor NF-Y is highly relevant for understanding PCa hallmarks. Unbalanced NF-YAs/NF-YAl ratio is associated not only to localized adenocarcinoma, but also to metastatic PCa that eventually develops resistance to therapies. Evaluation of the expression of NF-YA isoforms may be adopted as a new strategy for risk stratification of PCa patients and can be useful for defining biological properties of PCa (Fig. 9).

## Supplementary Information

**Additional file 1: Suppl. Figure S1. **Effects of NF-YA inactivation by RNAi *in vitro* and *in vivo*. (A) Cell proliferation curve measured by MTT assay of PC3 cells infected with scramble (shCTR) and NF-YA-targeting shRNA (shNF-YA). Data represent mean ± SEM (multiple t-test corrected by the Holm-Sidak method: *p<0.05, **p<0.01, n=4). (B) Images of xenograft tumors dissected from SCID Hairless Outbred (SHO®) mice after 5 weeks from s.c. injection of shCTR and shNF-YA cells (7 mice per group).

**Additional file 2: Suppl. Figure S2.** Effects of the overexpression of NF-YA isoforms in PCa cell lines. (A) Validation of the overexpression of NF-YAl and NF-YAs by western blot of whole cell extracts from LNCaP stable cell lines. Tubulin was used as loading control. (B) Colony number of Empty, NF-YAl and NF-YAs LNCaP cells cultured in anchorage-dependent growth condition. Data represent mean ± SEM (one-way ANOVA with Fisher's LSD test: *p<0.05, ***p<0.001, n=3). (C) Colony number of Empty, NF-YAl and NF-YAs LNCaP cells cultured in anchorage-independent growth condition. Data represent mean ± SEM (one-way ANOVA with Fisher's LSD test: *p<0.05, ns, not significant, n=6). (D) Time course analysis of cellular growth of LNCaP cultured as MTSs, calculated as projected area fold change relative to day 2, arbitrarily set at 1. Data represent mean ± SEM (two-way ANOVA with Holm-Sidak's test: *p<0.05, **p<0.01, ***p<0.001, ****p<0.0001, n=3). (E) Western blot analysis of NF-YA expression in Empty, NF-YAl and NF-YAs DU145 stable cell lines. Tubulin was used as loading control. (F) Time course analysis of cellular growth of DU145 cultured as MTSs, calculated as projected area fold change relative to day 4, arbitrarily set at 1. Data represent mean ± SEM (two-way ANOVA with Holm-Sidak's test: *p<0.05, **p<0.01, n=4). (G) Western blot of total extracts from PC3 MTSs with the indicated antibodies. Tubulin has been used as loading control.

**Additional file 3: Suppl. Figure S3.** CRISPR/Cas9-mediated knock out of endogenous *hNF-YA*. (A) Schematic representation of CRISPR/Cas9 strategy to knock down human *NF-YA*. The picture illustrates the sgRNA targeting endogenous *hNF-YA* (blue line) and the PAM sequence specific for the human gene (red line). Sequence alignment of the reverse complementary strand of exon 2 of human and mouse *NF-YA* gene is shown. (B) Indel spectrum determined by TIDE analysis on human *NF-YA* gene in a representative experiment of bulk CRISPR-treated PC3 cells overexpressing murine NF-YAl (left panel) or NF-YAs (right panel). Editing frequencies are shown (p < 0.05). (C) Indel spectrum determined by TIDE analysis on murine *NF-YA* in CRISPR-treated and GFP-sorted PC3 cells overexpressing murine NF-YAl (left panel) or NF-YAs (right panel). (D) Indel spectrum determined by TIDE analysis on human NF-YA gene in PC3 NF-YAs clone #11 (left panel) or #23 (right panel). Frequencies of editing are reported (p < 0.05) and show biallelic editing.

**Additional file 4: Suppl. Figure S4. **Gene signature of MTSs overexpressing NF-YAs or NF-YAl. Top 25 enriched GO terms of down regulated genes in NF-YAl (left panel) and NF-YAs (right panel) overexpressing PC3 MTSs vs Empty control ones. The size of each circle represents the number of genes included in each GO term and the color of the circle indicates the adjusted p value.

**Additional file 5: Suppl. Figure S5. **Unique gene signature of MTSs overexpressing NF-YAs or NF-YAl. (A) Top 25 enriched GO terms of up and down regulated genes in NF-YAl overexpressing cells vs Empty cells by setting |Log2FC| >1 and discarding genes with |Log2FC| >0.5 in NF-YAs vs Empty. (B) Top 25 enriched GO terms of up and down regulated genes in NF-YAs overexpressing cells vs Empty cells by setting |Log2FC|>1 and discarding genes with |Log2FC| >0.5 in NF-YAl vs Empty. The size of each circle represents the number of genes enriched in each GO term. Color bar indicates adjusted p-value for the labeled GO term.

**Additional file 6: Suppl. Figure S6.** Effect of NF-YA overexpression on tumor growth *in vivo* and analysis of NF-YA isoforms in PCa metastatic sites. (A) Volumes (mm^3^) of Empty, NF-YAs and NF-YAl xenograft tumors at the indicated time points. Data represent mean ± SEM (two-way ANOVA with Holm-Sidak's test: *p<0.05, **p<0.01, ns, not significant, n=8). (B) Images of xenograft tumors dissected from SCID Hairless Outbred (SHO®) mice after 5 weeks from s.c. injection. (C) Analysis of NF-YAs and NF-YAl transcripts in metastatic sites from PCa samples (GEO147250) and TCGA dataset (Prostate primary).

**Additional file 7: Table S1.** Oligonucleotides used in the experiments.

## Data Availability

All data supporting the conclusion of this article are included within the article and its additional files (Supplementary Figs. S1, S2, S3, S4, S5 and S6 and Supplementary Table S1). RNA-seq data are available in the Gene Expression Omnibus (GEO) repository, https://www.ncbi.nlm.nih.gov/geo (accession number GSE179990).

## Abbreviations

PCa: Prostate cancer
ECM: Extracellular matrix
EMT: Epithelial to mesenchymal transition
MET: Mesenchymal to epithelial transition
ADT: Androgen deprivation therapy
LUAD: Lung squamous adenocarcinomas
LUSC: Lung squamous cell carcinomas
EC: Endometrial cancer
TCGA: The cancer genome atlas
GS: Gleason score
CRPC: Metastatic castration-resistant prostate cancer
SCNC: Small cell neuroendocrine carcinoma
BPH: Benign hyperplasia
s.c.: Sub-cutaneous
MTS: Multicellular tumor spheroid
BrdU: Bromodeoxyuridine
H&E: Hematoxylin and eosin
GO: Gene ontology
RRHO: Rank-rank hypergeometric overlap
CTCs: Circulating tumor cells
FFPE: Formalin-fixed paraffin-embedded
