# Supplementary figures and images for: Alternative splicing of NF-YA promotes prostate cancer aggressiveness and represents a new molecular marker for clinical stratification of patients
Source: J Exp Clin Cancer Res. 2021 Nov 15;40:362. doi: 10.1186/s13046-021-02166-4 (PMC8594157; doi:10.1186/s13046-021-02166-4)

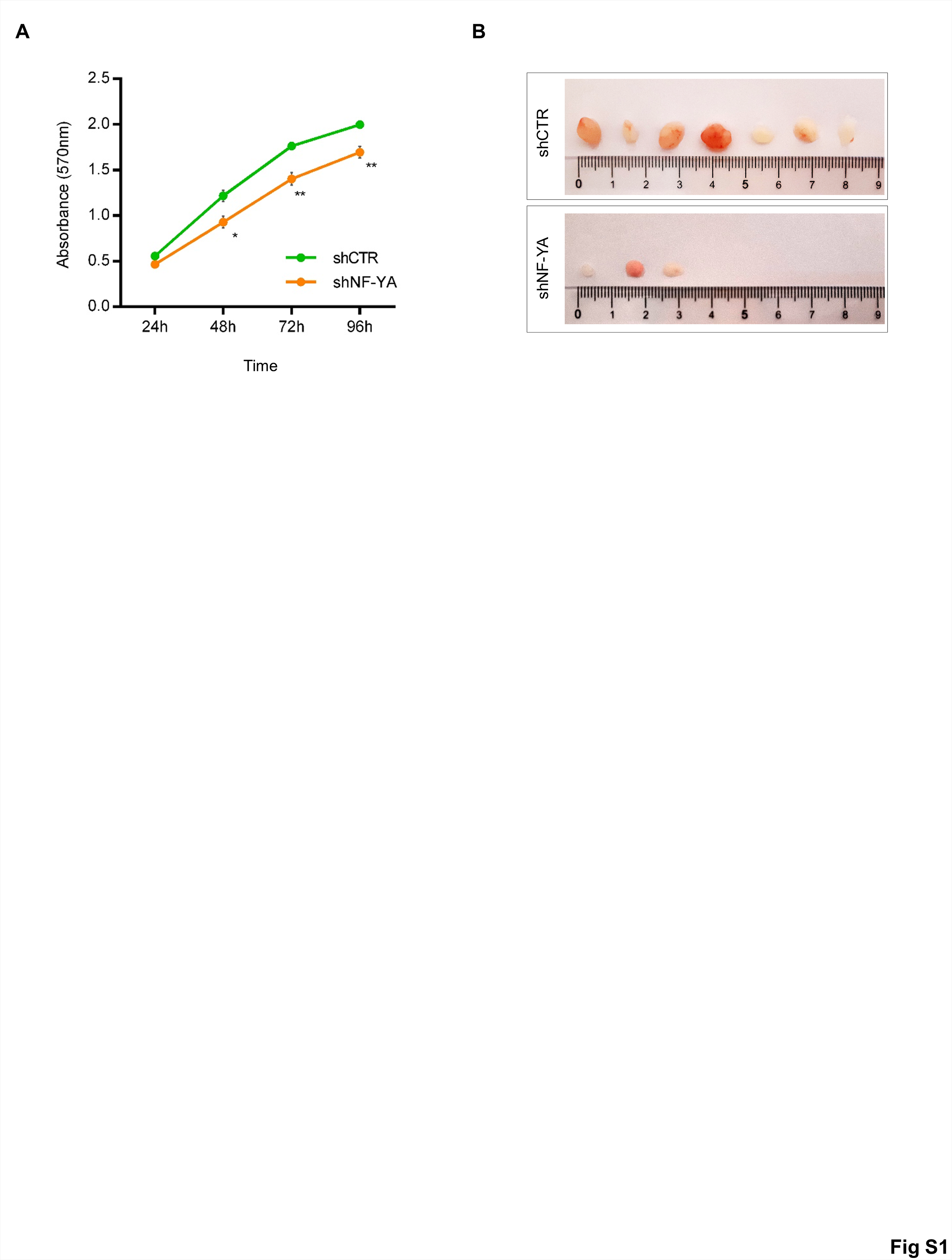

Supplement: Supplementary file 1 — Additional file 1: Suppl. Figure S1. Effects of NF-YA inactivation by RNAi in vitro and in vivo. (A) Cell proliferation curve measured by MTT assay of PC3 cells infected with scramble (shCTR) and NF-YA-targeting shRNA (shNF-YA). Data represent mean ± SEM (multiple t-test corrected by the Holm-Sidak method: *p<0.05, **p<0.01, n=4). (B) Images of xenograft tumors dissected from SCID Hairless Outbred (SHO®) mice after 5 weeks from s.c. injection of shCTR and shNF-YA cells (7 mice per group). [file 13046_2021_2166_MOESM1_ESM.tif]

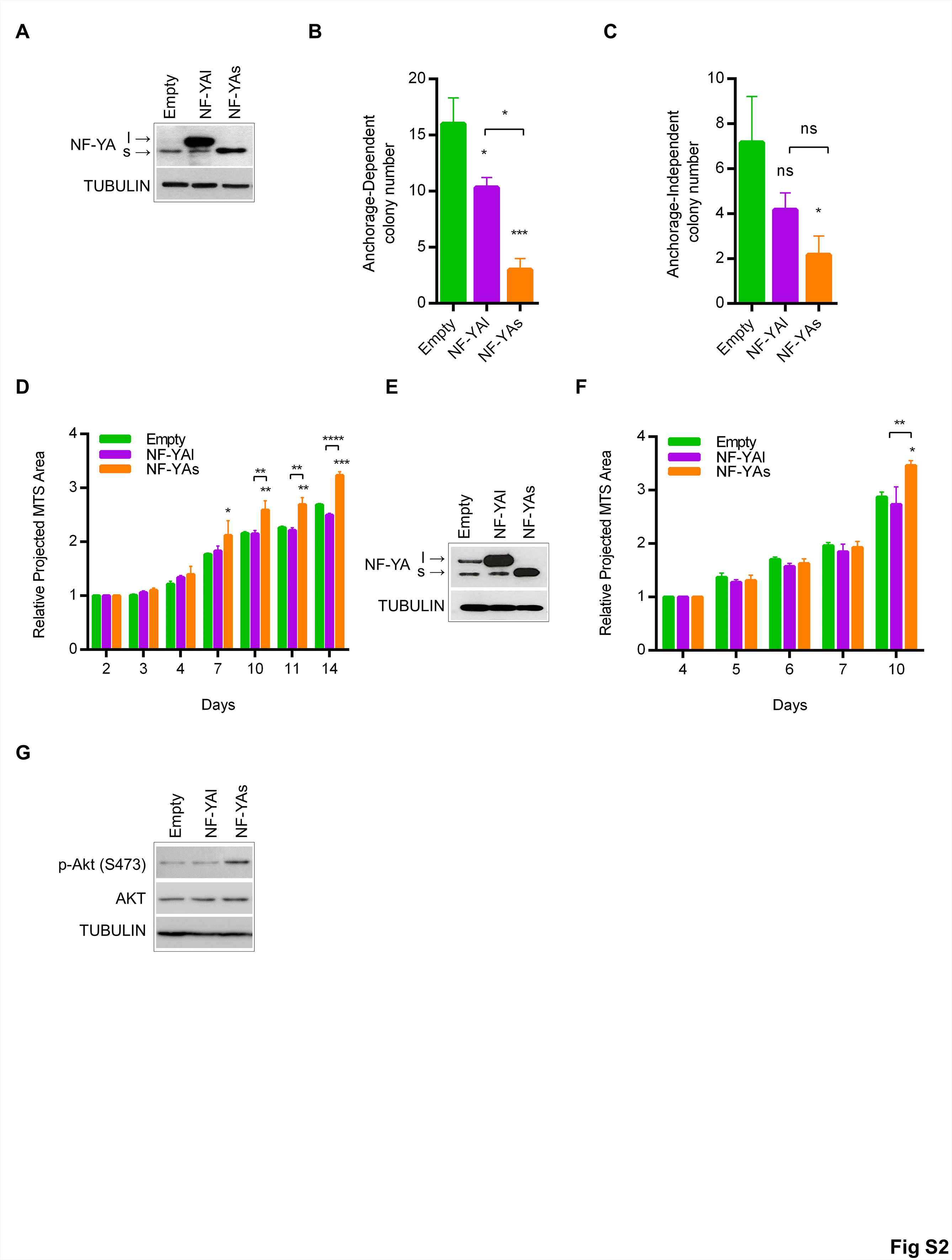

Supplement: Supplementary file 2 — Additional file 2: Suppl. Figure S2. Effects of the overexpression of NF-YA isoforms in PCa cell lines. (A) Validation of the overexpression of NF-YAl and NF-YAs by western blot of whole cell extracts from LNCaP stable cell lines. Tubulin was used as loading control. (B) Colony number of Empty, NF-YAl and NF-YAs LNCaP cells cultured in anchorage-dependent growth condition. Data represent mean ± SEM (one-way ANOVA with Fisher's LSD test: *p<0.05, ***p<0.001, n=3). (C) Colony number of Empty, NF-YAl and NF-YAs LNCaP cells cultured in anchorage-independent growth condition. Data represent mean ± SEM (one-way ANOVA with Fisher's LSD test: *p<0.05, ns, not significant, n=6). (D) Time course analysis of cellular growth of LNCaP cultured as MTSs, calculated as projected area fold change relative to day 2, arbitrarily set at 1. Data represent mean ± SEM (two-way ANOVA with Holm-Sidak's test: *p<0.05, **p<0.01, ***p<0.001, ****p<0.0001, n=3). (E) Western blot analysis of NF-YA expression in Empty, NF-YAl and NF-YAs DU145 stable cell lines. Tubulin was used as loading control. (F) Time course analysis of cellular growth of DU145 cultured as MTSs, calculated as projected area fold change relative to day 4, arbitrarily set at 1. Data represent mean ± SEM (two-way ANOVA with Holm-Sidak's test: *p<0.05, **p<0.01, n=4). (G) Western blot of total extracts from PC3 MTSs with the indicated antibodies. Tubulin has been used as loading control. [file 13046_2021_2166_MOESM2_ESM.tif]

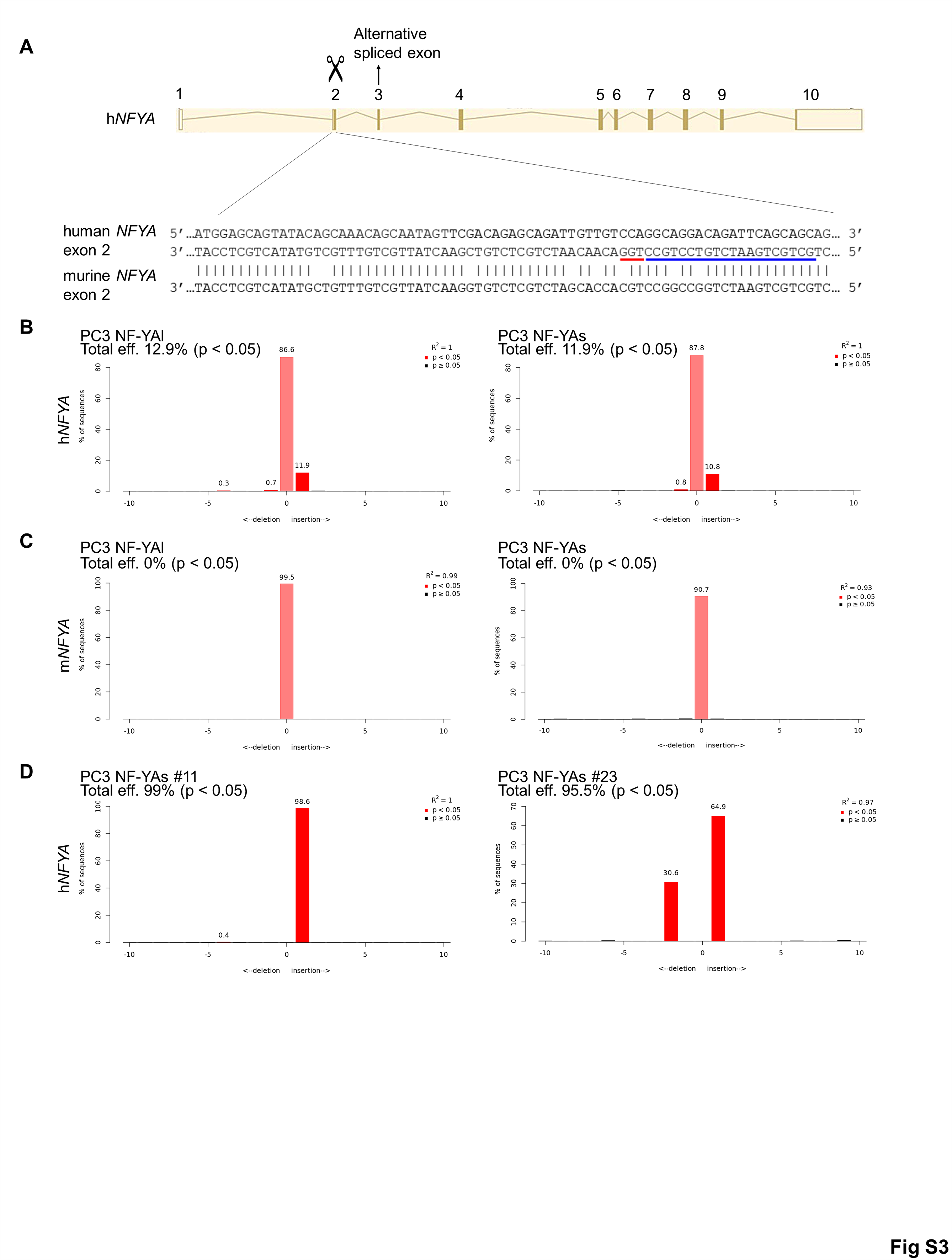

Supplement: Supplementary file 3 — Additional file 3: Suppl. Figure S3. CRISPR/Cas9-mediated knock out of endogenous hNF-YA. (A) Schematic representation of CRISPR/Cas9 strategy to knock down human NF-YA. The picture illustrates the sgRNA targeting endogenous hNF-YA (blue line) and the PAM sequence specific for the human gene (red line). Sequence alignment of the reverse complementary strand of exon 2 of human and mouse NF-YA gene is shown. (B) Indel spectrum determined by TIDE analysis on human NF-YA gene in a representative experiment of bulk CRISPR-treated PC3 cells overexpressing murine NF-YAl (left panel) or NF-YAs (right panel). Editing frequencies are shown (p < 0.05). (C) Indel spectrum determined by TIDE analysis on murine NF-YA in CRISPR-treated and GFP-sorted PC3 cells overexpressing murine NF-YAl (left panel) or NF-YAs (right panel). (D) Indel spectrum determined by TIDE analysis on human NF-YA gene in PC3 NF-YAs clone #11 (left panel) or #23 (right panel). Frequencies of editing are reported (p < 0.05) and show biallelic editing. [file 13046_2021_2166_MOESM3_ESM.tif]

# GO Terms DOWN: NF-YAI vs Empty

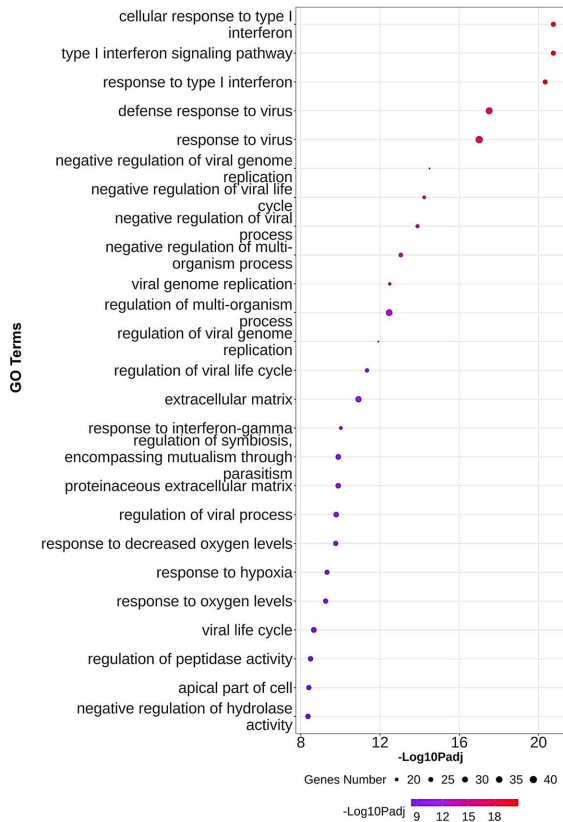

# GO Terms DOWN: NF-YAs vs Empty

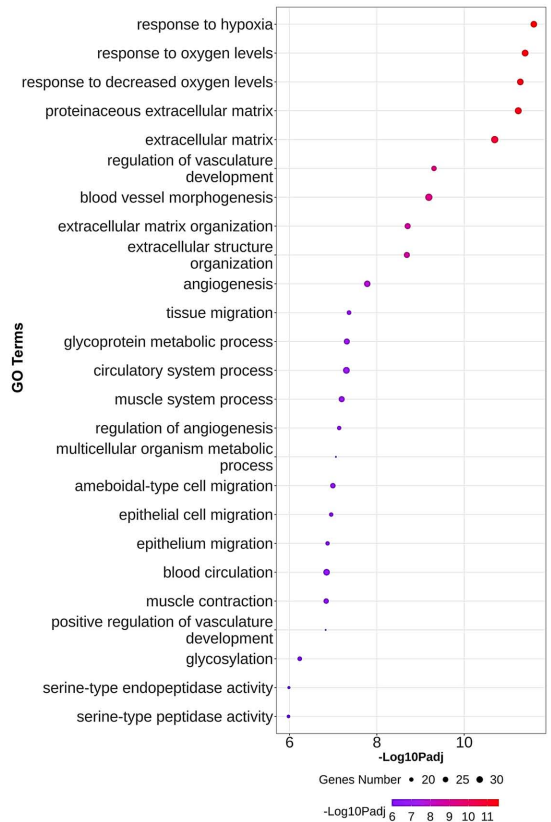

Supplement: Supplementary file 4 — Additional file 4: Suppl. Figure S4. Gene signature of MTSs overexpressing NF-YAs or NF-YAl. Top 25 enriched GO terms of down regulated genes in NF-YAl (left panel) and NF-YAs (right panel) overexpressing PC3 MTSs vs Empty control ones. The size of each circle represents the number of genes included in each GO term and the color of the circle indicates the adjusted p value. [file 13046_2021_2166_MOESM4_ESM.pdf]

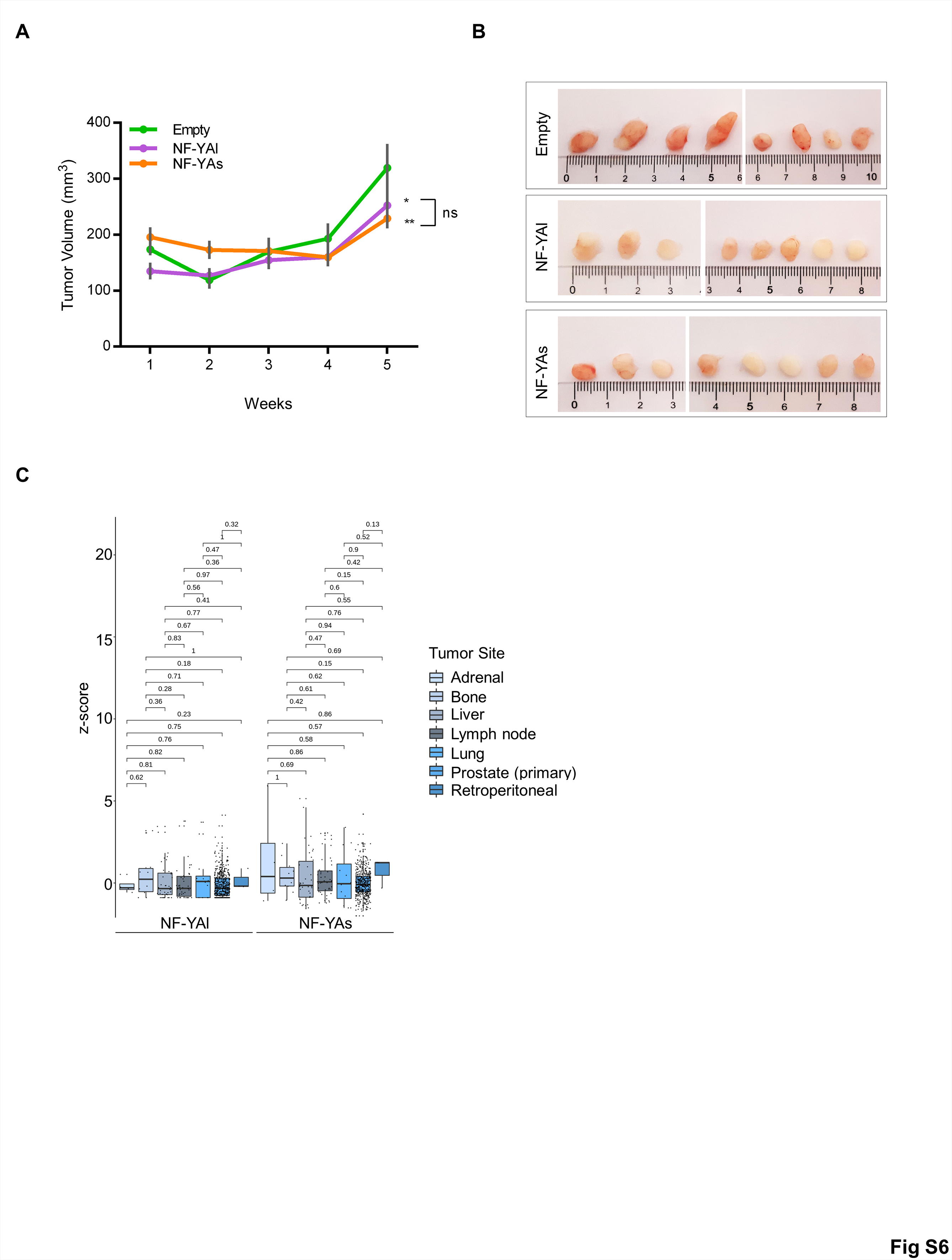

Supplement: Supplementary file 6 — Additional file 6: Suppl. Figure S6. Effect of NF-YA overexpression on tumor growth in vivo and analysis of NF-YA isoforms in PCa metastatic sites. (A) Volumes (mm3) of Empty, NF-YAs and NF-YAl xenograft tumors at the indicated time points. Data represent mean ± SEM (two-way ANOVA with Holm-Sidak's test: *p<0.05, **p<0.01, ns, not significant, n=8). (B) Images of xenograft tumors dissected from SCID Hairless Outbred (SHO®) mice after 5 weeks from s.c. injection. (C) Analysis of NF-YAs and NF-YAl transcripts in metastatic sites from PCa samples (GEO147250) and TCGA dataset (Prostate primary). [file 13046_2021_2166_MOESM6_ESM.tif]
