# Supplementary material for: Alternative splicing of NF-YA promotes prostate cancer aggressiveness and represents a new molecular marker for clinical stratification of patients
Source: J Exp Clin Cancer Res. 2021 Nov 15;40:362. doi: 10.1186/s13046-021-02166-4 (PMC8594157; doi:10.1186/s13046-021-02166-4)
Supplement: Supplementary file 5 — Additional file 5: Suppl. Figure S5. Unique gene signature of MTSs overexpressing NF-YAs or NF-YAl. (A) Top 25 enriched GO terms of up and down regulated genes in NF-YAl overexpressing cells vs Empty cells by setting |Log2FC| >1 and discarding genes with |Log2FC| >0.5 in NF-YAs vs Empty. (B) Top 25 enriched GO terms of up and down regulated genes in NF-YAs overexpressing cells vs Empty cells by setting |Log2FC|>1 and discarding genes with |Log2FC| >0.5 in NF-YAl vs Empty. The size of each circle represents the number of genes enriched in each GO term. Color bar indicates adjusted p-value for the labeled GO term. [file 13046_2021_2166_MOESM5_ESM.pdf]

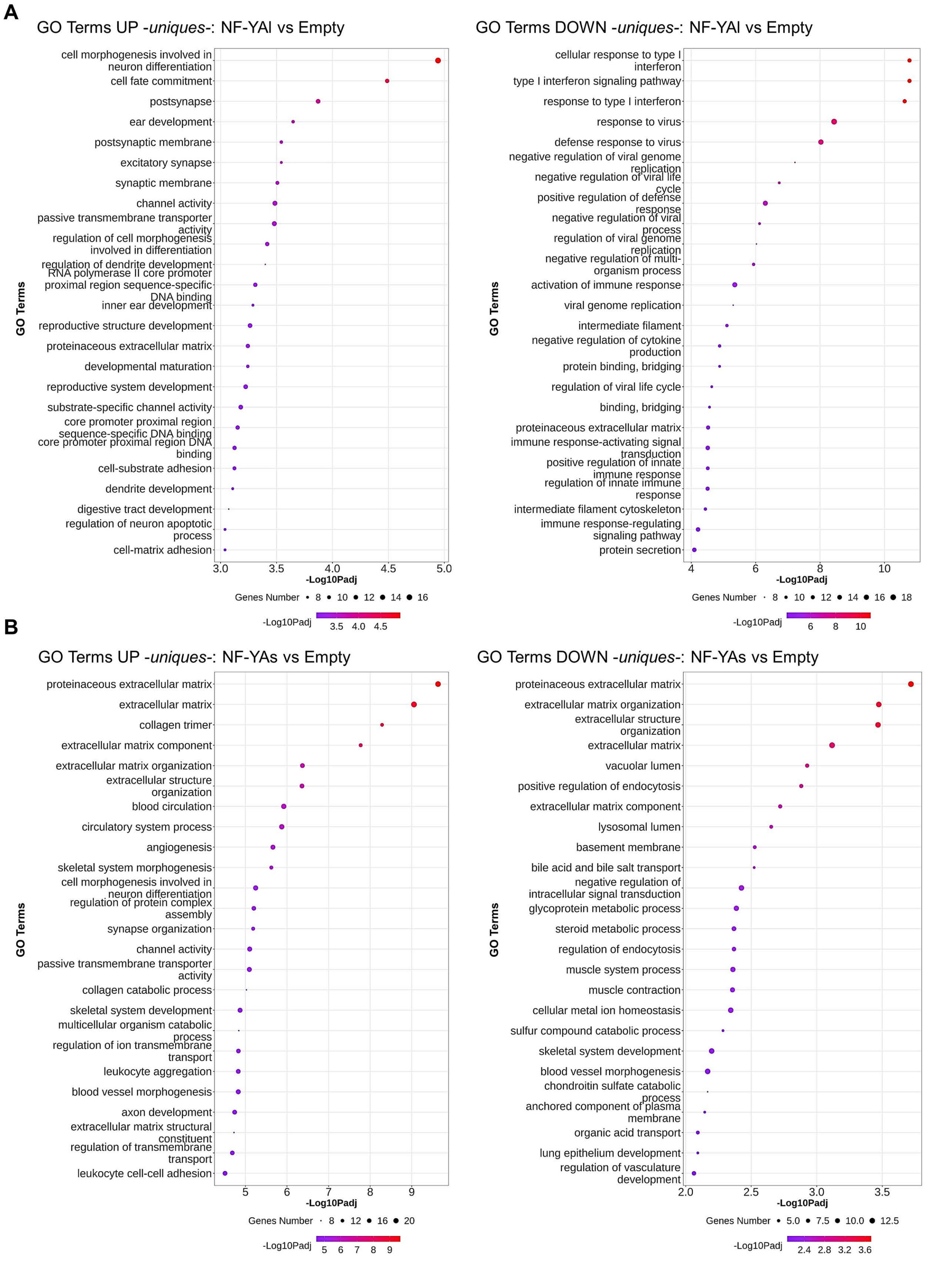
A
GO Terms UP -uniques-: NF-YAl vs Empty
cell morphogenesis involved in neuron differentiation
cell fate commitment
postsynapse
ear development
postsynaptic membrane
excitatory synapse
synaptic membrane
channel activity
passive transmembrane transporter activity
regulation of cell morphogenesis involved in differentiation
regulation of dendrite development
RNA polymerase II core promoter proximal region sequence-specific DNA binding
inner ear development
reproductive structure development
proteinaceous extracellular matrix
developmental maturation
reproductive system development
substrate-specific channel activity
core promoter proximal region sequence-specific DNA binding
core promoter proximal region DNA binding
cell-substrate adhesion
dendrite development
digestive tract development
regulation of neuron apoptotic process
cell-matrix adhesion
GO Terms
-Log10Padj
Genes Number 8 10 12 14 16
GO Terms DOWN -uniques-: NF-YAl vs Empty
cellular response to type I interferon
type I interferon signaling pathway
response to type I interferon
response to virus
defense response to virus
negative regulation of viral genome replication
negative regulation of viral life cycle
positive regulation of defense response
negative regulation of viral process
regulation of viral genome replication
negative regulation of multi-organism process
activation of immune response
viral genome replication
intermediate filament
negative regulation of cytokine production
protein binding, bridging
regulation of viral life cycle
binding, bridging
proteinaceous extracellular matrix
immune response-activating signal transduction
positive regulation of innate immune response
regulation of innate immune response
intermediate filament cytoskeleton
immune response-regulating signaling pathway
protein secretion
Genes Number 8 10 12 14 16 18
B
GO Terms UP -uniques-: NF-YAs vs Empty
proteinaceous extracellular matrix
extracellular matrix
collagen trimer
extracellular matrix component
extracellular matrix organization
extracellular structure organization
blood circulation
circulatory system process
angiogenesis
skeletal system morphogenesis
cell morphogenesis involved in neuron differentiation
regulation of protein complex assembly
synapse organization
channel activity
passive transmembrane transporter activity
collagen catabolic process
skeletal system development
multicellular organism catabolic process
regulation of ion transmembrane transport
leukocyte aggregation
blood vessel morphogenesis
axon development
extracellular matrix structural constituent
regulation of transmembrane transport
leukocyte cell-cell adhesion
Genes Number 8 12 16 20
GO Terms DOWN -uniques-: NF-YAs vs Empty
proteinaceous extracellular matrix
extracellular matrix organization
extracellular structure organization
extracellular matrix
vacuolar lumen
positive regulation of endocytosis
extracellular matrix component
lysosomal lumen
basement membrane
bile acid and bile salt transport
negative regulation of intracellular signal transduction
glycoprotein metabolic process
steroid metabolic process
regulation of endocytosis
muscle system process
muscle contraction
cellular metal ion homeostasis
sulfur compound catabolic process
skeletal system development
blood vessel morphogenesis
chondroitin sulfate catabolic process
anchored component of plasma membrane
organic acid transport
lung epithelium development
regulation of vasculature development
Genes Number 5.0 7.5 10.0 12.5

Fig S5
